# Dysregulation of microRNAs and tRNA-derived ncRNAs in mesothelial and mesothelioma cell lines after asbestiform fiber exposure

**DOI:** 10.1038/s41598-022-13044-0

**Published:** 2022-06-02

**Authors:** Veronica Filetti, Alessandro La Ferlita, Antonio Di Maria, Venera Cardile, Adriana C. E. Graziano, Venerando Rapisarda, Caterina Ledda, Alfredo Pulvirenti, Carla Loreto

**Affiliations:** 1 Human Anatomy and Histology Unit, Department of Biomedical and Biotechnological Sciences, University of Catania, 95123 Catania, Italy; 2 Bioinformatics Unit, Department of Clinical and Experimental Medicine, University of Catania, 95123 Catania, Italy; 3 Department of Cancer Biology and Genetics, James Cancer Center, The Ohio State University, Columbus, OH USA; 4 Physiology Unit, Department of Biomedical and Biotechnological Sciences, University of Catania, 95123 Catania, Italy; 5 Occupational Medicine, Department of Clinical and Experimental Medicine, University of Catania, 95123 Catania, Italy

**Keywords:** Cancer, Computational biology and bioinformatics, Biomarkers, Health occupations

## Abstract

Experimental evidence demonstrated that fluoro-edenite (FE) can develop chronic respiratory diseases and elicit carcinogenic effects. Environmental exposure to FE fibers is correlated with malignant pleural mesothelioma (MPM). An early diagnosis of MPM, and a comprehensive health monitoring of the patients exposed to FE fibers are two clinical issues that may be solved by the identification of specific biomarkers. We reported the microRNA (miRNA) and transfer RNA-derived non coding RNA (tRNA-derived ncRNA) transcriptome in human normal mesothelial and malignant mesothelioma cell lines exposed or not exposed to several concentration FE fibers. Furthermore, an interactive mesothelioma-based network was derived by using NetME tool. In untreated condition, the expression of miRNAs and tRNA-derived ncRNAs in tumor cells was significantly different with respect to non-tumor samples. Moreover, interesting and significant changes were found after the exposure of both cells lines to FE fibers. The network-based pathway analysis showed several signaling and metabolic pathways potentially involved in the pathogenesis of MPM. From papers analyzed by NetME, it is clear that many miRNAs can positively or negatively influence various pathways involved in MPM. For the first time, the analysis of tRNA-derived ncRNAs molecules in the context of mesothelioma has been made by using in vitro systems. Further studies will be designed to test and validate their diagnostic potential in high-risk individuals' liquid biopsies.

## Introduction

Malignant pleural mesothelioma (MPM) is an aggressive and rare malignant neoplasm of the pleural surface, predominantly caused by asbestos fibers exposure^1^. A high incidence of MPM due to asbestos exposure has been reported by several international studies conducted in Finland^2^, California, USA^3^, China^4^, Corsica^5^, New Caledonia^6^, Cyprus^7^, and Greece^8^. Literature also shows a high incidence of this malignancy caused by asbestiform fibers exposure^9^. Fluoro-edenite (FE) fibers fall into this category, along with erionite, antigorite, winchite, magnesio‑riebeckite, richterite, and Libby asbestos^9^. FE is a silicate mineral, an amphibole found in 1997 in Biancavilla, a small town of the Etnean volcanic complex (Sicily, Italy)^10^. This mineral presents some characteristics similar to the asbestos group^11^ and scientific evidence led to the classification of FE as Group 1 human carcinogens^12^.

Epidemiological studies have indeed confirmed that FE fibers have shown similar effects to those already reported after exposure to asbestos fibers^13–17^, leading to chronic inflammation, DNA damage, and carcinogenesis.

Thus, environmental exposure to these carcinogen fibers continues to represent a public health problem due to the long latency of MPM and to its aggression not alerted by specific symptoms. Certainly, the prevention of diseases related to carcinogen fibers exposure is to reduce their presence in the environment through reclamation, encapsulation, and confinement^9^. However, having available biomarkers that give information on the health state of the patients exposed to FE fibers or that allow an early diagnosis on MPM patients still asymptomatic would be a tremendous goal. Some studies have been conducted deep into the link between common genetic variations in the molecular pathways, and cancer risk in order to find useful biomarkers for screening and early diagnosis of MPM in fibers-exposed subjects. Interestingly, literature has demonstrated that non-coding RNAs (ncRNAs) may be used both as valuable non-invasive diagnostic and prognostic biomarkers and as therapeutic targets for cancer^18,19^.

As it is widely known, ncRNAs are a very heterogeneous class of RNA molecules that do not encode for proteins, and they represent a considerable amount of the transcriptome. More importantly, they are involved in several aspects of cell physiology by regulating a broad spectrum of cellular processes, from regulating gene expression to contributing to genome organization and stability^20,21^. ncRNAs are usually classified according to their size in small ncRNAs (< 200 nucleotides) and long ncRNAs (> 200 nucleotides)^20,21^. Alternatively, they can also be classified according to their function in housekeeping and regulatory ncRNAs^20,21^. The latter includes several classes of small RNA molecules in which microRNAs (miRNAs) and the new emergent transfer RNA-derived non-coding RNAs (tRNA-derived ncRNAs) represent very interesting components^20,21^.

Concerning miRNAs, they are long 18–25 nucleotide (nt) single-stranded RNAs, evolutionarily conserved, which negatively modulate the expression of their target messenger RNAs (mRNAs). They bind to the 3′ Untranslated Region (3′ UTR) of specific mRNA targets, leading to translational repression, or mRNA cleavage^21^. miRNAs are very important molecules in the regulation of gene expression at the post-transcriptional level, indeed, a single miRNA can control the expression of several mRNAs and a single mRNA may be targeted by more than one miRNA, thus creating a complex network of cooperative regulation^21^. Since the discovery of miR-15\16 deletion in Chronic lymphocytic leukemia (CLL) patients in 2002^22^, many studies have been published about miRNAs and their involvement in the pathogenesis of several human cancers^23^. In addition, many efforts have been made to use miRNAs present in biological fluids as non-invasive diagnostic and prognostic biomarkers for several human cancers^24^. Unfortunately, despite the extensive work, very few miRNAs might be used today in clinical practice^24^.

Contrary to miRNAs, tRNA-derived ncRNAs are a very heterogeneous class of ncRNAs that derive from tRNA processing. Indeed, in the last few years, several kinds of tRNA-derived ncRNAs have been discovered. However, a unique classification is still missing. A common grouping of such molecules is based on the location they originate from within the tRNA gene. Therefore, tRNA-derived ncRNAs can be divided into two main classes: (i) tRNA-derived small RNAs (tsRNAs), which derive from pre-tRNA; (ii) and tRNA-derived fragments (tRFs), which derive from mature tRNA^25^. tsRNAs are produced inside the nucleus and result from the cleavage of the pre-tRNAs 3’ trailer sequence by ribonucleases Z (RNases Z). They usually begin after the 3’-end of mature tRNAs and are characterized by a polyuracil sequence at their 3’-ends^25^. On the other hand, tRFs, ranging from 14–30 nt in length, are derived from mature tRNA^26–28^. Precisely, tRF-5 s are generated in the cytoplasm by Dicer-mediated cleavage of the mature tRNA D-loop^29,30^ while tRF-3 s are produced in the cytoplasm via cleavage of the T-loop in mature tRNAs operated by Dicer, angiogenin, and other members of the ribonuclease A (RNase A) superfamily. They are fragments originating from mature tRNA 3’-ends and include the final CCA sequence^28,29,31^. All these tRNA-derived ncRNA classes have been recently identified to have a major role in cancer biology. Indeed, it has been shown that tRNA-derived ncRNAs are not mere byproducts of random tRNA cleavage, rather they may actively play roles in several biological phenomena such as ribosome biogenesis, retrotransposition, virus infections, apoptosis, and cancer pathogenesis^25,32–40^. Furthermore, some classes of tRNA-derived ncRNAs have been shown to bind argonaute (AGO) and PIWI proteins, potentially acting as post- or pre-transcriptional regulators of gene expression^37,41^. Accumulating evidence also suggests the presence of functional tRNA-derived ncRNAs in human biological fluids, such as urine and serum from cancer patients^26,42–45^. However, such molecules have never been analyzed in the context of mesothelioma.

**Figure 1:**
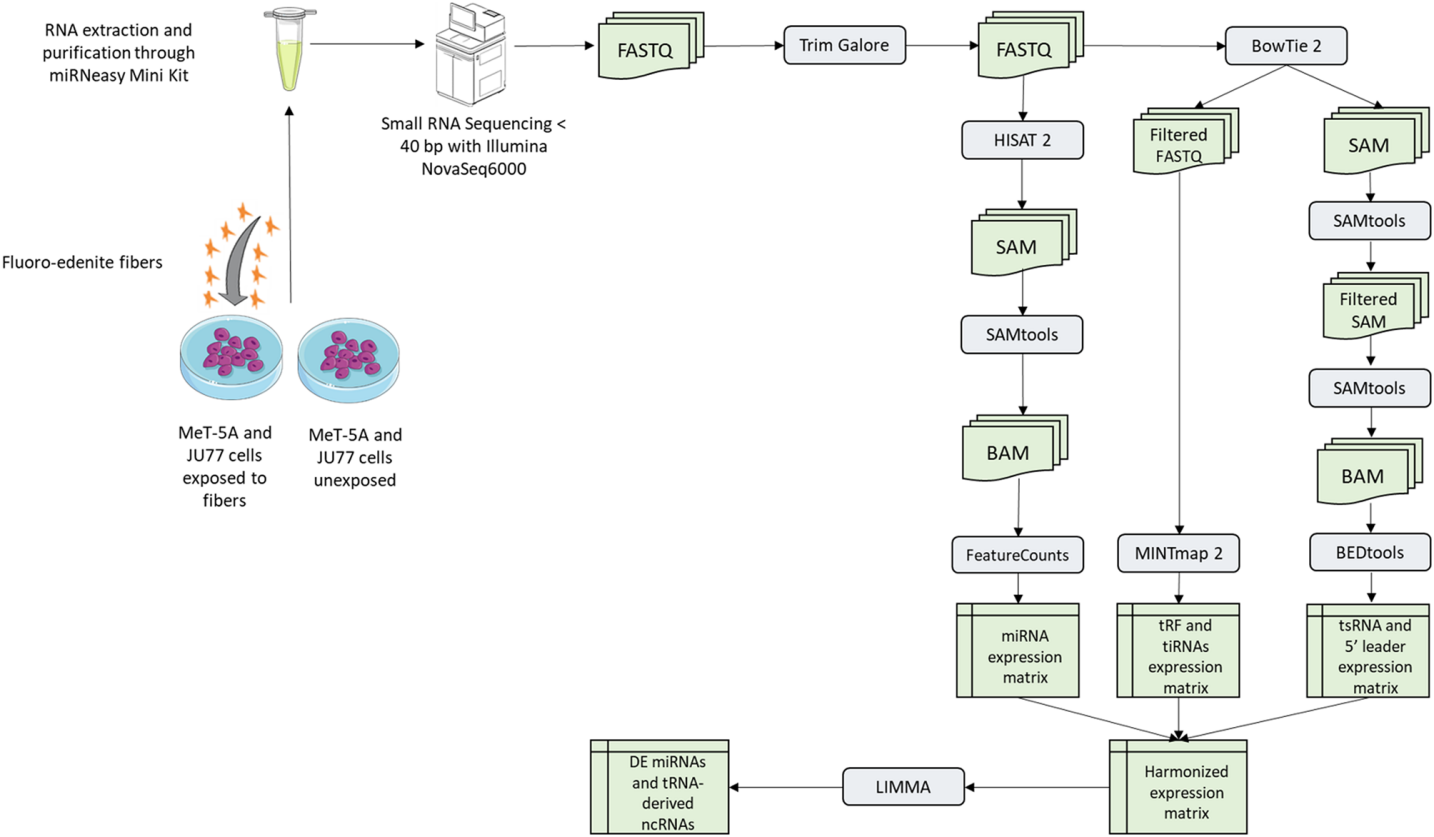
Experimental workflow used to extract, process, and analyze RNA from cell lines.

As mentioned above, in this study, we reported for the first time a small RNA-Seq transcriptome profiling of healthy mesothelial and malignant mesothelioma cell lines exposed to FE fibers. Precisely, we analyzed the miRNA and tRNA-derived ncRNA transcriptome in a human normal mesothelial cell line (MeT-5A) and in a human malignant mesothelioma cell line (JU77). Both these cell lines have been processed with and without fluoro-edenite fibers exposure, as reported in the experimental workflow (Fig. 1). All cell lines characteristics and the functional in vitro experiments were shown in Table 1.

**Table 1:** Characteristics of cell lines and functional in vitro experiments.

Cell line	Species	Origin	Morphology	Treatment	Exposure time
MeT-5A	Homo sapiens	Healthy mesothelium	Epithelial-like	/	/
MeT-5A	Homo sapiens	Healthy mesothelium	Epithelial-like	10 µg/ml fluoro-edenite fibers	48 h
MeT-5A	Homo sapiens	Healthy mesothelium	Epithelial-like	50 µg/ml fluoro-edenite fibers	48 h
JU77	Homo sapiens	Malignant mesothelioma	Epithelial-like	/	/
JU77	Homo sapiens	Malignant mesothelioma	Epithelial-like	10 µg/ml fluoro-edenite fibers	48 h
JU77	Homo sapiens	Malignant mesothelioma	Epithelial-like	50 µg/ml fluoro-edenite fibers	48 h

Furthermore, in this study, a mesothelioma-based knowledge graph has been derived from PubMed central^46^ full texts (17,762 full texts) by using NetME tool^47^. The utility of this graph is to allow scientists, biologists, and researchers to find or learn logical relationships/interactions between MPM and other biological and chemical entities (such as miRNAs, diseases, xenobiotics, etc.) without the need to download and read mesothelioma-based articles available in the literature. The knowledge graph is released in a csv format and could be loaded in Neo4j^48^ or Cytoscape^49^ to be queried.

## Results

### Differential expression analysis

**Figure 2:**
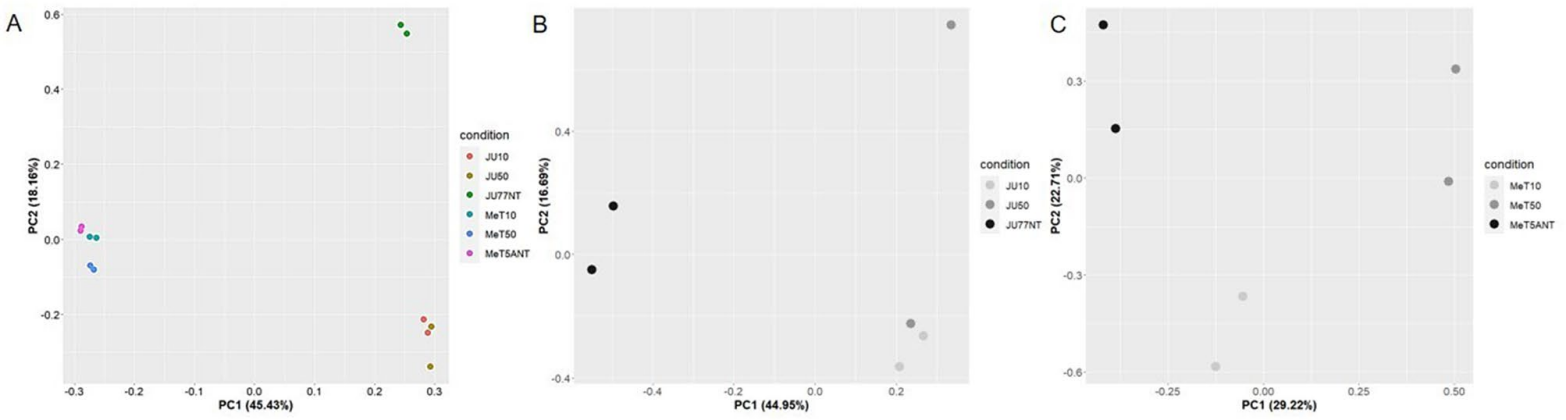
Principal Component Analysis (PCA) showing a separation in miRNAs and tRNA-derived ncRNAs between: (**A**) JU77 (human malignant mesothelioma) vs. MeT-5A (human normal mesothelium) cell lines; (**B**) JU77 controls vs. exposed to 10 and 50 ug/ml FE fibers; (**C**) MeT-5A controls vs. exposed to 10 and 50 ug/ml FE fibers.

In order to identify dysregulation in miRNAs and tRNA-derived ncRNAs induced by FE fibers, we performed an RNA-Seq transcriptome profiling of unexposed and exposed normal mesothelial (MeT-5A) and malignant mesothelioma (JU77) cell lines. Differential expression analysis of such data showed major differences in small ncRNAs expression. Specifically, differences were observed when we compared MeT-5A and JU77 unexposed vs. FE-exposed at the same concentration. The Principal Component Analysis (PCA) showed a separation between MeT-5A and JU77 cell lines in their miRNAs and tRNA-derived ncRNAs expression. The greater separation was observed between the two different cell lines (PC1: 45.43%) while treated and untreated cell lines showed minor differences (PC2: 18.16%) (Fig. 2A). Moreover, to better highlight intra-cell line differences in small ncRNA expression, we performed PCAs specifically for each cell line (Figs. 2B, C). These confirmed a great separation between treated and untreated JU77 (PC1: 44.95%) (Fig. 2B). As about MeT-5A cells, the separation was greater by comparing untreated cells with that one exposed to 50 µg/ul of FE fibers (PC1: 29.22%) (Fig. 2C). In combination, these results seem to suggest that although differences in small ncRNA expression between treated and untreated MeT-5A were detected, major effects were observed in the JU77 cell line.

**Figure 3:**
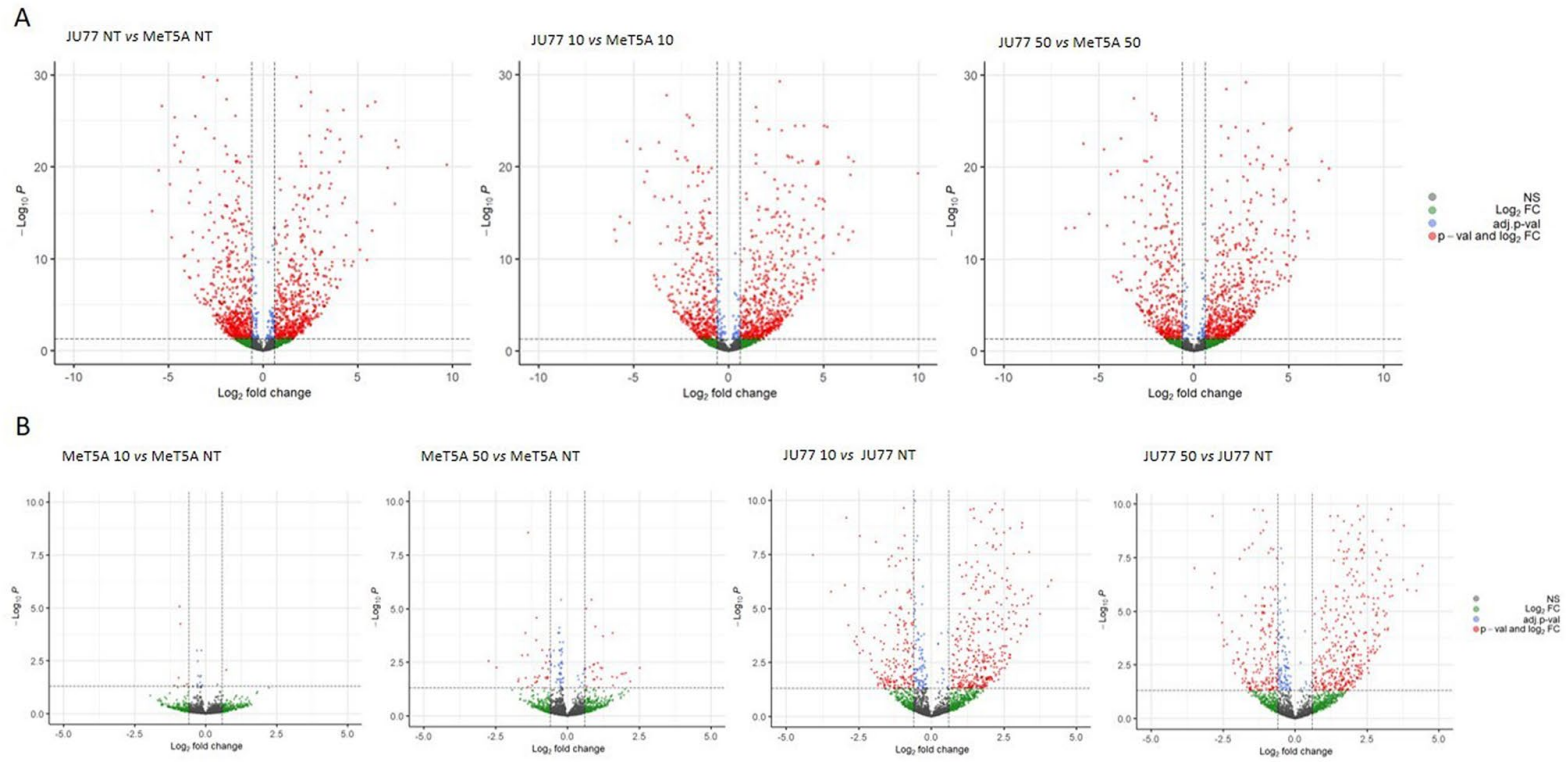
Volcano plots showing the differentially expressed miRNAs and tRNA-derived ncRNAs between: (**A**) JU77 (human malignant mesothelioma) vs. MeT-5A (human normal mesothelium); (**B**) Untreated vs. FE fibers treated JU77 and MeT-5A.

The differentially expressed miRNAs and tRNA-derived ncRNAs were: 1171 in untreated JU77 vs. MeT-5A, 960 in JU77 vs. MeT-5A exposed to 10 ug/ml FE fibers, and 969 in JU77 vs. MeT-5A exposed to 50 ug/ml FE fibers (Fig. 3A). The common population of differentially expressed miRNAs and tRNA-derived ncRNAs between the two cell lines increased with the exposure to FE fibers.

**Figure 4:**
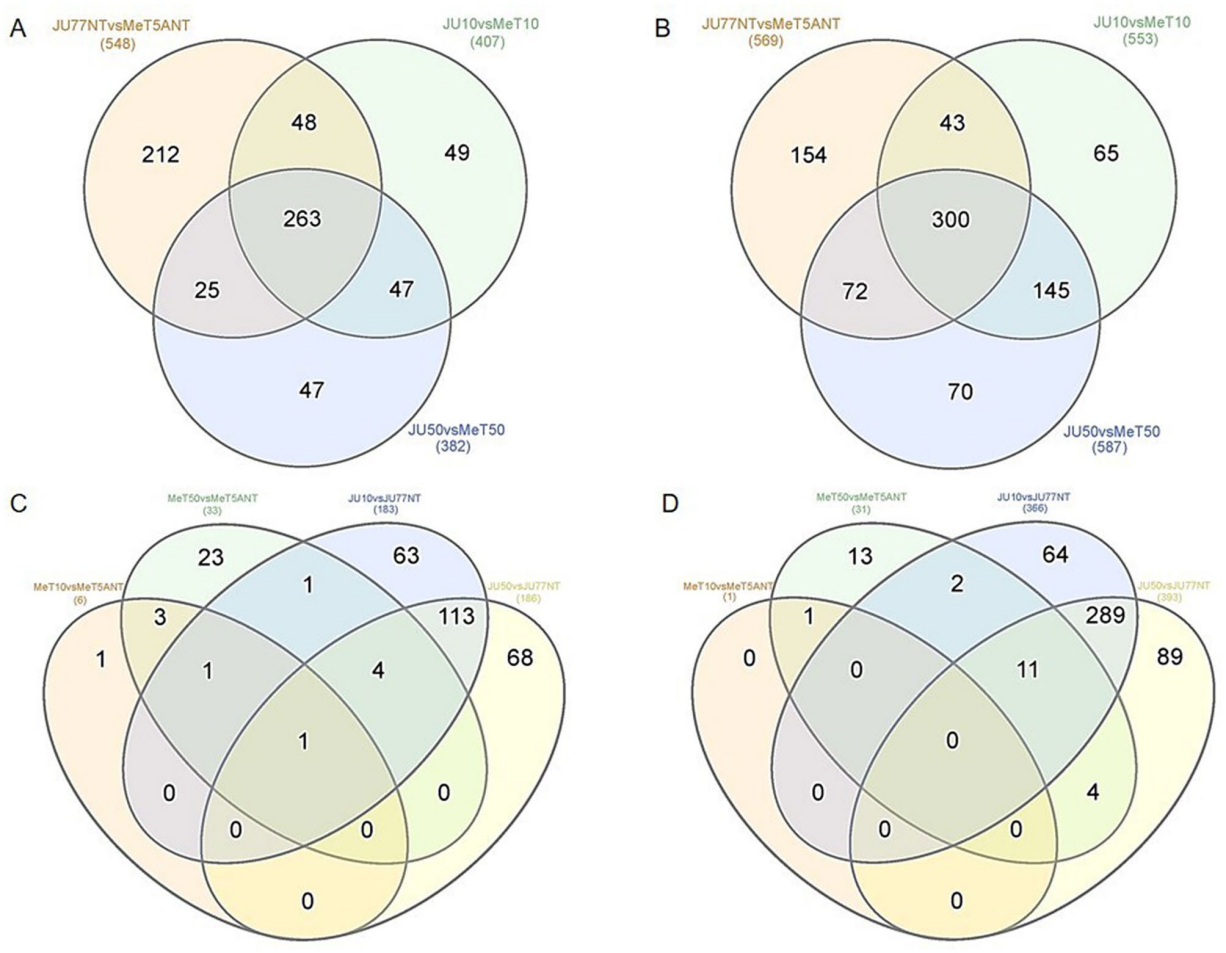
Venn diagrams showing down-regulated miRNAs and tRNA-derived ncRNAs distribution between: (**A**) JU77 vs. MeT-5A cell lines both not exposed and exposed to FE fibers; (**C**) The same cellular line both not exposed and exposed to FE fibers. Venn diagrams showing up-regulated miRNAs and tRNA-derived ncRNAs distribution between: (**B**) JU77 vs. MeT-5A cell lines both not exposed and exposed to FE fibers; (**D**) The same cellular line both not exposed and exposed to FE fibers.

In more detail, the down-regulated miRNAs and tRNA-derived ncRNAs were: 548 in untreated JU77 vs. MeT-5A, 407 in JU77 vs. MeT-5A exposed to 10 ug/ml FE fibers, and 382 in JU77 vs. MeT-5A exposed to 50 ug/ml FE fibers (Fig. 4A). Among all samples, the differentially expressed miRNAs and tRNA-derived ncRNAs in common were 263 (Fig. 4A). Among these, 48 were in common between the JU77 and MeT-5A untreated and exposed to 10 ug/ml FE fibers (40 tRNA-derived ncRNAs and 8 miRNAs), 25 were in common between the JU77 and MeT-5A untreated and exposed to 50 ug/ml FE fibers (22 tRNA-derived ncRNAs and 3 miRNAs), and 47 were in common between the JU77 and MeT-5A exposed to 10 and 50 ug/ml FE fibers (30 tRNA-derived ncRNAs and 17 miRNAs) (Fig. 4A, Table 2).

**Table 2:** Down-regulated ncRNAs in common among JU77 (human malignant mesothelioma) vs. MeT-5A (human normal mesothelium) controls and JU77 vs. exposed to 10 and 50 ug/ml FE fibers; down-regulated ncRNAs in common among JU77 vs. MeT-5A exposed to 10 and exposed to 50 ug/ml FE fibers.

Down-regulated ncRNAs in common		
JU77NTvs.MeT5ANT and JU10vs.MeT10	JU77NTvsMeT5ANT and JU50vsMeT50	JU10vsMeT10 and JU50vsMeT50
tRF-43-ZMB3UEVPQOH7HLWXD2	tRF-26-VJUE3QHMY1B	tRF-18-8R6Q46D2
tRF-34-XRK4WO2F9IY9E2	tRF-26-MJ8F81KLB5D	ts-52
tRF-27-5BF900BY4D5	tRF-30-M2OSRNLNKSEK	tRF-18-H5KQBFD2
tRF-33-XRK4WO2F9IY9V	tRF-27-HK4B8XVQN52	tRF-17-08Q2B52
5P_tRNA-His-GTG-1–1	tRF-23-QOH7HLWXD2	tRF-19-J9BBF3E2
tRF-17-9N1EWJM	tRF-40-ZSE6YBY4HEJQY1B6	tRF-35-4S14IZJQXEXOIS
tRF-31-QKF1R3WE8RO80	tRF-17-73V2Y8K	tRF-28-4S14IZJQXE0M
tRF-35-I1KQSX0DIJZ726	tRF-21-941QKS3WD	tRF-22-WB86N7O52
tRF-28-PS5P4PW3FJDD	tRF-20-9DH48V7K	tRF-29-4S14IZJQXEJU
tRF-39-H2SBUL6JYBKRYNJO	tRF-16-XXW27ZE	tRF-22-WEKSPM852
tRF-17-BS68BFJ	tRF-41-SO1XDXVJUE3QHMY1B	tRF-23-Z5EFOK8YDZ
tRF-19-V47P59I8	tRF-48-9NZ6V6Z3M8ZLSSXUOLD2	tRF-19-1SS2PMER
tRF-33-M2OSRNLNKSEKDS	tRF-19-DRVQHZE2	tRF-32-3JVIJMRPFQ5D5
tRF-34-QKF1R3WE8RO8IS	5P_tRNA-Val-AAC-2–1	tRF-31-YSV4V4SQ2WW1E
tRF-30-FN8DYDZDL9X1	tRF-18-J6K6UDV	tRF-22-8EKSP1852
tRF-35-1WZ4RZDMKNR7F6	tRF-19-Y87HFKJJ	tRF-22-8B8SOUPR2
tRF-36-D4ZWRNU3KQ9MV1B	tRF-18-H7HLWXD2	tRF-20-L3KQNKWH
tRF-36-9LVPZO0BEBXNWDE	tRF-24-ROWFUBNO0W	tRF-17-73V6M7M
tRF-18-VKS4I7D4	tRF-17-2YU04DQ	tRF-22-WB8647O52
tRF-31-Y2MJ8F81KLB5D	tRF-28-PLBIUBNXJMX	tRF-31-WZVLUE15MXE9D
tRF-37-U37Y93Y086Z34J2	tRF-16-S8V0J80	tRF-18-I6BB5LD2
tRF-29-32VIJMRPFQFX	tRF-18-9LVPZOU	tRF-26-32VIJMRPFQD
tRF-24-FSXMSL732Z		tRF-24-L3KZM8WM2Q
tRF-20-Q622EVUI	hsa-miR-4645-3p	tRF-19-9DH48V24
tRF-20-S8V0J8O9	hsa-miR-33a-3p	tRF-20-BLS17ONR
tRF-17-KE6KM8N	hsa-miR-2355-5p	tRF-18-18VUY9DW
tRF-20-NB8PLML3		tRF-30-7RH3S3RX8HWV
tRF-45-EZ5P1OHE10NO0RNQYX		tRF-22-WB0Q37Q52
tRF-22-W087W4SV2		tRF-20-L3KZM8WM
tRF-37-7O58J0K8UMPLBIO		tRF-27-LEK5YY0FYWQ
tRF-45-BF82Z4D7OOJ0QXL13X		
tRF-27-INVDRI2Q2R2		hsa-miR-22-3p
tRF-17-8R6Q46J		hsa-miR-140-3p
tRF-37-9LVPZO0BEBXNWD5		hsa-miR-381-3p
tRF-16-3KJBP1B		hsa-miR-132-5p
tRF-23-86V8WPMNY		hsa-miR-3176
tRF-22-WKXU53K8N		hsa-miR-323a-3p
tRF-16-4P64R90		hsa-miR-296-5p
tRF-25-V4V4SQ2WW1		hsa-let-7 g-3p
tRF-41-U5YKFN8DYDZDL9X1B		hsa-miR-19b-3p_1
		hsa-miR-32-3p
hsa-miR-1248		hsa-miR-378a-5p
hsa-miR-26a-5p_1		hsa-miR-15a-3p
hsa-miR-324-5p		hsa-miR-548 k
hsa-miR-1301-3p		hsa-miR-7974
hsa-miR-664a-3p		hsa-miR-671-3p
hsa-miR-641		hsa-miR-561-3p
hsa-miR-3651		hsa-miR-7-5p_1
hsa-miR-627-3p		

As for the up-regulated miRNAs and tRNA-derived ncRNAs, these were: 569 in untreated JU77 vs. MeT-5A, 553 in JU77 vs. MeT-5A exposed to 10 ug/ml FE fibers, and 587 in JU77 vs. MeT-5A exposed to 50 ug/ml FE fibers (Fig. 4B). Among all samples, the differentially expressed miRNAs and tRNA-derived ncRNAs in common were 300 (Fig. 4B). Among these, 43 were in common between the JU77 and MeT-5A untreated and exposed to 10 ug/ml FE fibers (37 tRNA-derived ncRNAs and 6 miRNAs), 72 were in common between the JU77 and MeT-5A untreated and exposed to 50 ug/ml FE fibers (54 tRNA-derived ncRNAs and 18 miRNAs), and 145 were in common between the JU77 and MeT-5A exposed to 10 and 50 ug/ml FE fibers (143 tRNA-derived ncRNAs and 2 miRNAs) (Fig. 4B, Table 3).

**Table 3:** Up-regulated ncRNAs in common among JU77 (human malignant mesothelioma) vs. MeT-5A (human normal mesothelium) controls and JU77 vs. exposed to 10 and 50 ug/ml FE fibers; down-regulated ncRNAs in common among JU77 vs. MeT-5A exposed to 10 and exposed to 50 ug/ml FE fibers.

Up-regulated ncRNAs in common					
JU77NTvsMeT5ANT and JU10vsMeT10	JU77NTvsMeT5ANT and JU50vsMeT50		JU10vsMeT10 and JU50vsMeT50		
ts-9	tRF-33-79MP9P9MH57SD3	hsa-miR-4521	tRF-17-8WQ0D52	tRF-37-LP21W3WB8US5652	tRF-19-1R7HFEE2
ts-7	tRF-33-PSQP4PW3FJIKW	hsa-miR-92a-3p_1	tRF-16-KQ3SW1B	tRF-26-63U0EZY9X1B	tRF-30-JMRPFQ5DWXH9
tRF-19-LNKSEKHY	tRF-40-70K8HJ83ML5F82NZ	hsa-miR-532-5p	tRF-19-N2NYDRE8	tRF-26-MI7O3B1NR8E	tRF-20-K87SIRM1
tRF-32-5BVNUEVBWXQZ4	tRF-32-MIF91SS2P46I3	hsa-miR-429	tRF-41-8OYDVNZDBQ8BB2S1B	tRF-34-I8W47W1R7HFEE2	tRF-21-987X1RJY0
tRF-16-ML5HMVE	tRF-33-MIF91SS2P4FIDQ	hsa-miR-485-3p	tRF-31-R008R959KUMKB	tRF-34-34HWH3RXSINHKN	tRF-36-L85DMKYUYRLHR0D
tRF-16-ML5F82D	ts-17	hsa-miR-30c-2-3p	tRF-33-PS5P4PW3FJHPW	tRF-17-WS72092	tRF-19-PS5P4PJ4
tRF-18-I8W47WZ	tRF-20-L3K5J0WB	hsa-miR-493-3p	tRF-42-Y6IO43ZU2FYOB0152	tRF-25-5NB2NZW7O6	tRF-35-EO7N987X1RJYSX
ts-11	tRF-31-5BVNUEVBWXQZ0	hsa-miR-29b-2-5p	tRF-33-Z3M8ZLSSXUOLD2	tRF-32-7EMQ18Y3E7QNN	tRF-20-Y8NNBSBK
tRF-22-WE8SP6X52	tRF-33-3JVIJMRPFQRD03	hsa-miR-190b	tRF-42-YDU37Y93Y086Z34J2	tRF-30-623K7SIR3DR2	tRF-33-RRJ89O9NF5W8W
tRF-26-9NS334L2H1B	tRF-18-W3FJHPW	hsa-miR-502-3p	tRF-17-8SPOL52	tRF-40-2VR0PSR9593J2426	tRF-26-M0IBB7Z92KB
tRF-25-6IZB8PLML3	tRF-20-LIK898OH	hsa-miR-532-3p	tRF-30-945NB2NZW7O6	tRF-17-8S68L52	tRF-39-86J8WPMN1E8Y7ZFV
tRF-16-RPM830E	tRF-39-70K8HJ83ML5F82HQ	hsa-miR-500a-3p	tRF-17-KSP1852	tRF-31-PW5SVP9N15WV0	tRF-26-U3XOUZDZN1B
tRF-31-3JVIJMRPFQRDE	tRF-30-MIF91SS2P4FI	hsa-miR-409-5p	tRF-20-KS7SB1RH	tRF-19-Z3M8ZL2J	tRF-43-Z6V6Z3M8ZLSSXUOLD2
tRF-22-LEKPMK3WP	tRF-36-PSQP4PW3FJI0E7E	hsa-miR-431-3p	tRF-43-Y6FPZD3KYUYR66EFD2	tRF-17-WJ9X0UF	tRF-27-90YBOZZ7ND2
tRF-25-LEKPV03X1N	tRF-22-45N2P4Z9Q	hsa-miR-660-3p	tRF-40-XENDBP1IUUK7VZ0R	tRF-20-4VZ87HFK	tRF-26-Q6S8V0J6OZE
tRF-19-FEWS3VF8	tRF-20-PSQP4PW3	hsa-miR-505-5p	tRF-41-XENDBP1IUUK7VZ0RB	tRF-18-YR66EFD2	tRF-37-Y91293WEW6VM4J2
tRF-30-MIF91SS2P46I	tRF-16-O0SRMND	hsa-miR-502-5p	tRF-23-ZOSRI8WPD2	tRF-37-87R8WP9N1EWJQ72	tRF-18-Y9PYKHD4
tRF-24-739P8WQ0EB	tRF-21-U9XW983F0	hsa-miR-2277-5p	tRF-17-8Y3HSV2	ts-89	tRF-24-7W1R7HFEE2
tRF-28-F91SS2PMFI0Q	tRF-36-87R8WP9N1EWJQ7D		tRF-27-945NB2NZW7L	tRF-34-MUWLV47PU9XWK0	tRF-21-XOUZDZN1B
tRF-19-S998LOJX	tRF-33-MIF91SS2P46IDQ		tRF-18-Y8NNBS6	tRF-29-34HWH3RXSIHM	tRF-26-727OFIZ9WUD
tRF-33-2QR7F8YKIR9N05	tRF-19-LE308HI1		tRF-17-KS8WP92	tRF-29-R9JP9P9NH523	tRF-39-86V8WPMN1E8Y7ZFV
tRF-16-XIW282B	tRF-29-2E489B3VI8KQ		tRF-35-PW5SVP9N15WV7W	tRF-26-08R959KUMKB	tRF-18-Y9H33PD2
tRF-30-3JVIJMRPFQ5D	tRF-41-70K8HJ83ML5F82NZB		tRF-29-945NB2NZW7HV	tRF-39-XENDBP1IUUK7VZEJ	tRF-16-K8M0W1B
tRF-22-3638FQ7Z3	tRF-20-S3M8309N		tRF-26-R81XDDZZ4YB	tRF-25-3K7SIR3DR2	tRF-37-L85DMKYUYRLHR0J
tRF-18-XIW282X	tRF-32-9M739P8WQ0D52		tRF-41-8HM2OSRNLNKSEK51B	tRF-32-PW5SVP9N15WVN	tRF-44-9XEO7N987X1RJYSXI2
tRF-32-6XQ6S8V0J8O9Q	tRF-38-EH623K7SIR3DR2DV		tRF-17-W18FLUF	tRF-16-WJ9X0UB	tRF-19-9N15WV2P
tRF-16-739P8W0	tRF-17-8R1546J		tRF-28-727OFIZ9WUD2	tRF-38-YDU37Y93Y086Z3DO	tRF-24-VUUMHNU6E2
tRF-26-17JOWU1M70E	tRF-22-8190JWZ75		tRF-34-PW5SVP9N15WV2P	tRF-16-8871K9D	tRF-18-SRI8WPD2
tRF-16-9NS334D	tRF-34-Y6V4VH7Q2WNIK1		tRF-20-WVZ5EFOK	tRF-18-0RSSX7D2	tRF-22-877343RX4
tRF-20-WJ9X0UD3	tRF-21-LEKPV03XB		5P_tRNA-His-GTG-1–8	tRF-33-7EMQ18Y3E7QN00	tRF-31-I363U0EZY9X1B
tRF-32-6LQ6S8V0J6OZQ	tRF-35-PS5P4PW3FJHPEZ		tRF-21-MI7O3B1N0	tRF-29-PSQP4PW3FJF4	tRF-20-7KZOSRI8
tRF-17-HO53KYN	tRF-34-Y6V4V47Q2WNIK1		tRF-36-M2OSRNLNKSEK51B	tRF-23-WJ9X0UD304	tRF-17-YR66EFJ
tRF-19-LNKSEKH9	tRF-36-Q99P9P9NH57S36D		tRF-35-EH623K7SIR3DR2	tRF-36-EO7N987X1RJYSX0	tRF-32-R9JP9P9NH5SY3
tRF-19-LJK1JKE4	tRF-30-R9JP9P9NH5SY		tRF-29-R81XDDZZ4YE2	tRF-36-XSXMSL73VL4YMYE	tRF-16-PSQP4PE
tRF-22-9M739P8WM	tRF-34-PSQP4PW3FJI0E5		tRF-40-KEUI1ZXF0N2BD0U6	tRF-17-W6VM4J2	tRF-37-PSQP4PW3FJIKE7O
tRF-17-7M3PVIK	tRF-20-LNK88KO4		tRF-42-YQHQ9M739P8WQ0D52	tRF-18-MI7O3BY	
tRF-18-QQKNR70H	tRF-17-OB9ZFH4		tRF-43-7O409Z7KZOSRI8WPD2	tRF-38-V6Z3M8ZLSSXUOLD2	hsa-miR-335-5p
	tRF-19-L61MQKKK		tRF-28-K8HJ83ML5F02	tRF-16-3VWIQ1B	hsa-miR-5586-5p
hsa-miR-376b-3p	tRF-16-YPSV17D		tRF-21-U0EZY9X1B	tRF-20-K87SERM4	
hsa-miR-188-5p	tRF-28-LE3V8FQMEP0Q		5P_tRNA-His-GTG-1–7	tRF-21-RNLNK88K0	
hsa-miR-410-3p	tRF-21-NRDF7UK80		tRF-28-R81XDDZZ4YV	tRF-17-RPM830K	
hsa-miR-29b-3p_1	tRF-25-8Q9KZR3HJ3		tRF-16-3VLIE1B	tRF-16-Q01LQKB	
hsa-miR-449a	tRF-34-KSLP7SDRXSE5I2		tRF-26-IQ5O8LZ521B	tRF-17-9NS334K	
hsa-miR-548u	tRF-35-JYSWRYVMMV5BU6		tRF-24-1XDDZZ4YE2	tRF-25-M0IBB7Z92K	
	tRF-40-OB9ZFH690M0RHB26		tRF-27-727OFIZ9WUJ	tRF-26-Z3M8ZLSSXU0	
	tRF-17-RN8RQF4		tRF-16-362VO00	tRF-20-387SDRJH	
	tRF-37-P4R8YP9LON4VN11		tRF-20-48Z8SSFK	tRF-20-J0DPZOIP	
	tRF-29-W47W1R7HFEE2		tRF-42-8OP3X1M3WE8SPOL52	tRF-39-9Q53K87SHRMF3RE2	
	tRF-25-SP58309MUK		tRF-27-93Y086Z34J2	tRF-18-XEKJ5RDS	
	5P_tRNA-Pro-AGG-2–1		tRF-17-86Z34J2	tRF-19-URYRSIE2	
	tRF-19-WJ9X0U0Y		tRF-23-1XDDZZ4YV	tRF-39-XU53F85SI1LQ3RE2	
	tRF-32-897PVP9N1QKSJ		tRF-33-PW5SVP9N15WV0E	tRF-38-WD8NBNIED6VZ0OD2	
	tRF-28-Z8XO46DODLD2		tRF-26-8DYDZDL9X1B	tRF-21-OB9ZFH69B	
	tRF-37-Q99P9P9NH57S362		tRF-34-M2OSRNLNKSEKH9	tRF-39-PSQP4PW3FJIKE727	

When we compared miRNAs and tRNA-derived ncRNAs expression between unexposed vs. exposed MeT-5A the results showed several differentially expressed molecules. In particular, miRNAs and tRNA-derived ncRNAs differentially expressed in MeT-5A untreated vs. exposed to 10 and 50 ug/ml FE fibers were 7 and 64, respectively (Fig. 3B). Indeed, the miRNAs and tRNA-derived ncRNAs differentially expressed in MeT-5A increased in dose-dependent manner with the exposure to FE fibers. On the other hand, the comparison between untreated vs. FE-treated JU77 showed a substantial difference between the differentially expressed miRNAs and tRNA-derived ncRNAs. In particular, miRNAs and tRNA-derived ncRNAs differentially expressed in JU77 unexposed vs. exposed to 10 and 50 ug/ml FE fibers were 549 and 579, respectively (Fig. 3B).

In more detail, the down-regulated miRNAs and tRNA-derived ncRNAs were: 6 and 33 in untreated MeT-5A vs. exposed to 10 and 50 ug/ml FE fibers, respectively (Fig. 4C). While in the case of neoplastic cells, the down-regulated miRNAs and tRNA-derived ncRNAs were: 183 and 186 in untreated JU77 vs. exposed to 10 and 50 ug/ml FE fibers, respectively (Fig. 4C). Among these, 3 were in common between the MeT-5A untreated and exposed to 10 ug/ml FE fibers vs. MeT-5A untreated and exposed to 50 ug/ml FE fibers (tRF-42-YDU37Y93Y086Z34J2, tRF-40-XENDBP1IUUK7VZ0R, and tRF-41-XENDBP1IUUK7VZ0RB), 113 were in common between the JU77 untreated and exposed to 10 ug/ml FE fibers vs. JU77 untreated and exposed to 50 ug/ml FE fibers (87 tRNA-derived ncRNAs and 26 miRNAs), only hsa-miR-3618 was in common between the MeT-5A untreated and exposed to 50 ug/ml FE fibers vs. JU77 untreated and exposed to 10 ug/ml FE fibers, no compound was in common between the MeT-5A untreated and exposed to 50 ug/ml FE fibers vs. JU77 untreated and exposed to 50 ug/ml FE fibers (Fig. 4C, Table 4). If we compare MeT-5A exposed to 10 ug/ml FE fibers vs. MeT5A untreated and MeT-5A exposed to 50 ug/ml FE fibers vs. MeT5A untreated and JU77 exposed to 10 ug/ml FE fibers vs. JU77 untreated the only compound in common was hsa-miR-1248 (Fig. 4C, Table 4). tRF-40-8B7OIQ1IBM4LV7U6, tRF-31-79MP9P9MH57SD, tRF-32-P4R8YP9LON4V3, tRF-25-SP58309MUK were the down-regulated tRNA-derived ncRNAs in common among MeT-5A exposed to 50 ug/ml FE fibers vs. MeT5A untreated and JU77 exposed to 10 ug/ml FE fibers vs. JU77 untreated and JU77 exposed to 50 ug/ml FE fibers vs. JU77 untreated (Fig. 4C, Table 4). Among all samples, tRF-33-79MP9P9MH57SD3 was the only down-regulated compound in common (Fig. 4C, Table 4).

**Table 4:** Down-regulated ncRNAs in common among JU77 (human malignant mesothelioma) vs. MeT-5A (human normal mesothelium) in all tested conditions.

Down-regulated ncRNAs in common			
MeT10*v*MeT5ANT and MeT50vs.MeT5ANT	JU10vs.JU77NT and JU50vs.JU77NT		
tRF-42-YDU37Y93Y086Z34J2	tRF-33-Q99P9P9NH57SD3	ts-7	tRF-35-86J8WPMN1E8Y7Z
tRF-40-XENDBP1IUUK7VZ0R	tRF-16-RPM830D	tRF-20-L3KQNKWH	tRF-22-WEK6S1852
tRF-41-XENDBP1IUUK7VZ0RB	ts-49	tRF-22-WE8SPOL52	tRF-16-R29P4PE
	tRF-34-Q99P9P9NH57S15	tRF-31-WZVLUE15MXE9D	tRF-19-1SS2PMER
MeT10vs.MeT5ANT and MeT50vs.MeT5ANT and JU10vs.JUNT	ts-111	tRF-17-W3FJHP1	tRF-31-YSV4V4SQ2WW1E
hsa-miR-1248	ts-52	tRF-20-L3KZM8WM	tRF-34-4WVLV470VR31KD
	tRF-35-LSM1M3WE8SSP6D	5P_tRNA-His-GTG-1–5	tRF-21-45N2P4Z9E
MeT10vs.MeT5ANT and MeT50vs.MeT5aNT and JU10vs.JU77NT and JU50vs.JU77NT	tRF-20-9P8WQ0D5	tRF-27-86J8WPMN1E5	tRF-20-LMK1JKE2
tRF-33-79MP9P9MH57SD3	ts-70	tRF-40-I6D3987SDR265M5Z	tRF-22-8FLUD3KSN
	tRF-22-9P8WQ0D52	5P_tRNA-Gln-TTG-3–3	ts-51
MeT50vs.MeT5ANT and JU10vs.JUNT	tRF-34-P4R8YP9LON4VHM	tRF-33-U4727OFIZ9WUD2	tRF-22-WEKSPM852
hsa-miR-3618	tRF-32-3JVIJMRPFQ5D5	tRF-34-K84J83ML5F92H3	
	ts-34	tRF-22-79MP9PMNI	hsa-let-7 g-3p
MeT50vsMeT5ANT and JU50vsJU77NT	tRF-22-1SS2PMFIQ	tRF-22-8EKSP1852	hsa-miR-200b-3p
-	tRF-33-79MP9PMNH5ISD3	tRF-22-WE8SPOX52	hsa-let-7c-3p
	tRF-19-S334L2F1	tRF-36-PSQP4PW3FJI0E7E	hsa-miR-7974
MeT50vsMeT5ANT and JU10vsJU77NT and JU50vsJU77NT	tRF-16-S3M830E	tRF-18-I8W47WZ	hsa-miR-100-3p
tRF-40-8B7OIQ1IBM4LV7U6	tRF-37-HQ9M739P8WQ0D52	tRF-18-I6BB5LD2	hsa-miR-503-5p
tRF-31-79MP9P9MH57SD	ts-98	tRF-23-JMRPFQJD0Q	hsa-miR-222-5p
tRF-32-P4R8YP9LON4V3	tRF-19-J9BBF3E2	tRF-20-H1NRM5U6	hsa-miR-484
tRF-25-SP58309MUK	ts-101	tRF-22-WE8SP6X52	hsa-miR-363-3p
	tRF-32-3JVIJMRPFQRD5	ts-112	hsa-miR-4521
	ts-96	tRF-32-5BVNUEVBWXQZ4	hsa-miR-29a-5p
	ts-20	tRF-16-ML5F82D	hsa-miR-18a-5p
	ts-2	tRF-31-MIF91SS2P46ID	hsa-miR-29b-1-5p
	tRF-40-70K8HJ83ML5F82NZ	tRF-31-3JVIJMRPFQ5DE	hsa-miR-18a-3p
	tRF-34-79MP9PMNH5IS15	tRF-16-I6D3880	hsa-miR-503-3p
	tRF-33-3JVIJMRPFQRD03	ts-95	hsa-miR-362-5p
	tRF-33-87R8WP9N1EWJDW	tRF-33-3JVIJMRPFQ5D03	hsa-miR-20a-3p
	ts-23	tRF-31-5BVNUEVBWXQZ0	hsa-let-7a-2-3p
	tRF-16-PJ5830E	tRF-42-D3IPBBKN8LEZSO1XF	hsa-miR-200a-3p
	tRF-28-4S14IZJQXE0M	tRF-22-WB86N7O52	hsa-miR-132-5p
	tRF-29-4S14IZJQXEJU	tRF-18-INVDRID1	hsa-miR-32-3p
	tRF-18-YDVNZDR	tRF-17-H7V3LYN	hsa-miR-125b-2-3p
	tRF-21-9P8WQ0D5D	tRF-35-86V8WPMN1E8Y7Z	hsa-miR-16–1-3p
	tRF-18-H5KQBFD2	tRF-20-W3FJHPEZ	hsa-miR-19b-1-5p
	tRF-33-PSQP4PW3FJIKW	tRF-32-MIF91SS2P46I3	hsa-miR-429
	ts-68	tRF-18-W3FJHPW	hsa-miR-548 k

As for the up-regulated miRNAs and tRNA-derived ncRNAs, these were: 1 and 31 in untreated MeT-5A vs. exposed to 10 and 50 ug/ml FE fibers, respectively (Fig. 4D). While in the case of neoplastic cells, the up-regulated miRNAs and tRNA-derived ncRNAs were: 366 and 393 in untreated JU77 vs. exposed to 10 and 50 ug/ml FE fibers, respectively (Fig. 4D). Among these, only ts-96 was in common between the MeT-5A untreated and exposed to 10 ug/ml FE fibers vs. MeT-5A untreated and exposed to 50 ug/ml FE fibers, 289 were in common between the JU77 untreated and exposed to 10 ug/ml FE fibers vs. JU77 untreated and exposed to 50 ug/ml FE fibers (among these only one was a miRNA, specifically hsa-miR-615-3p) (Fig. 4D, Table 5). tRF-16-PSSELQB, and tRF-21-JYSWRYVMD.

**Table 5:** Up-regulated ncRNAs in common among JU77 (human malignant mesothelioma) vs. MeT-5A (human normal mesothelium) in all tested conditions.

Up-regulated ncRNAs in common				
MeT10*v*MeT5ANT and MeT50vs.MeT5ANT	JU10vs.JU77NT and JU50vs.JU77NT			
ts-96	tRF-17-8WQ0D52	tRF-30-M2OSRNLNKSEK	tRF-34-MUWLV47PU9XWK0	tRF-18-Y8NNBS6
	tRF-16-3JWB61B	tRF-22-YBOZZ7ND2	tRF-36-L85DMKYUYRLHR0D	tRF-27-R81XDDZZ4Y1
MeT10vs.MeT5ANT and MeT50vs.MeT5ANT and JU10vs.JUNT	tRF-26-5BF900BY4DE	tRF-34-34HWH3RXSINHKN	tRF-42-8OP3X1M3WE8SPOL52	tRF-17-8Y3HSV2
-	tRF-32-897PVP941QKSJ	tRF-21-WSNSRV500	tRF-39-XENDBP1IUUK7VZEJ	tRF-19-J6K6UDE2
	tRF-16-K8KDP1B	tRF-25-5BF900BY4D	tRF-35-1WZ4RZDMKNR7F6	tRF-33-PS5P4PW3FJHPW
MeT10vs.MeT5ANT and MeT50vs.MeT5aNT and JU10vs.JU77NT and JU50vs.JU77NT	tRF-32-M2OSRNLNKSEKL	tRF-39-EO7N987X1RJYSXI2	tRF-26-JYSWRYVMMV0	tRF-30-945NB2NZW7O6
-	tRF-22-5BF900BY3	tRF-31-PW5SVP9N15WV0	tRF-22-WEW6VM4J2	tRF-21-WSNYRV500
	tRF-17-884U1D2	tRF-34-PW5SVP9N15WV2P	tRF-20-Y8NNBSBK	tRF-17-K6S1852
MeT50vs.MeT5ANT and JU10vs.JUNT	tRF-33-QKF1R3WE8RO8DX	tRF-18-Y9H33PD2	tRF-35-E6YBY4HEJQY1B6	tRF-23-ZOSRI8WPD2
tRF-16-PSSELQB	tRF-16-KQ3SW1B	tRF-23-8RR9ODMJDX	tRF-26-M0IBB7Z92KB	tRF-27-93Y086Z34J2
tRF-21-JYSWRYVMD	tRF-19-N2NYDRE8	tRF-35-PW5SVP9N15WV7W	tRF-18-HSQSD2D2	tRF-34-I8W47W1R7HFEE2
	tRF-16-L7P5QKB	tRF-38-V6Z3M8ZLSSXUOLD2	tRF-35-7OIQ1IBM4LV7U6	tRF-18-5BF900R
MeT50vsMeT5ANT and JU50vsJU77NT	tRF-41-8OYDVNZDBQ8BB2S1B	tRF-27-5BF900BY4D5	tRF-26-ROWFUBNOBZE	tRF-24-1XDDZZ4YE2
tRF-18-WKXU53DJ	tRF-17-88481D2	tRF-19-1R7HFEE2	tRF-16-2KWHR90	tRF-29-R81XDDZZ4YE2
tRF-48-9NZ6V6Z3M8ZLSSXUOLD2	tRF-33-86J8WPMN1E8Y0E	tRF-20-4VZ87HFK	tRF-46-7Z8L8NRS9NS334L2H1B	tRF-26-08R959KUMKB
tRF-19-OSM83OJX	tRF-16-884U1DD	tRF-32-QKF1R3WE8RO84	tRF-29-PS5P4PW3FJF2	tRF-17-KS8WP92
tRF-22-7OFIZ9WUJ	tRF-16-8WQ0D5D	tRF-17-8871K92	tRF-16-K8M0W1B	tRF-44-ZW20YBYL6XDRVQHZE2
	tRF-19-5BF9000W	tRF-19-Z3M8ZL2J	tRF-41-YDU37Y93Y086Z34JD	tRF-30-P4R8YP9LON4V
MeT50vsMeT5ANT and JU10vsJU77NT and JU50vsJU77NT	tRF-32-86J8WPMN1E8YN	tRF-24-VUUMHNU6E2	tRF-26-Z3V5393L41B	tRF-43-Y6FPZD3KYUYR66EFD2
tRF-28-727OFIZ9WUD2	tRF-28-945NB2NZW7DR	tRF-33-86V8WPMN1E8Y0E	tRF-27-MJ8F81KLB52	tRF-25-5NB2NZW7O6
tRF-18-51MLHVDB	tRF-16-K5J0W1B	tRF-22-4B8XVQN52	tRF-33-9Z7KZOSRI8WPD2	tRF-26-R81XDDZZ4YB
tRF-19-86J8WP1Z	tRF-17-K0SVRNK	tRF-31-VDRI2Q2RJO01B	tRF-17-941QKSJ	tRF-16-34L2H1B
tRF-18-7X9PN5D5	tRF-31-R008R959KUMKB	tRF-21-5BF900BYD	tRF-35-I1KQSX0DIJZ726	tRF-26-VJUE3QHMY1B
tRF-23-1XDDZZ4YV	tRF-39-X553387SHRJL3RE2	tRF-17-86Z34J2	tRF-26-727OFIZ9WUD	tRF-36-M2OSRNLNKSEK51B
tRF-19-7X9PN5HJ	tRF-20-5BF900BY	tRF-39-HMI8W47W1R7HFEE2	tRF-43-96L85DMKYUYRLHR0D2	tRF-17-863IP52
tRF-40-ZSE6YBY4HEJQY1B6	tRF-38-L85DMKYUYRLHR0D2	tRF-20-387SDRJH	tRF-26-90YBOZZ7NDD	tRF-40-KEUI1ZXF0N2BD0U6
tRF-20-48Z8SSFK	tRF-17-8689SV2	tRF-40-JQJYSWRYVMMV5BU6	tRF-31-I363U0EZY9X1B	tRF-43-ZMB3UEVPQOH7HLWXD2
tRF-31-897PVP941QKSD	tRF-33-Z3M8ZLSSXUOLD2	tRF-27-90YBOZZ7ND2	tRF-32-ZPEK45H5KQBFJ	tRF-29-945NB2NZW7HV
tRF-18-J6K6UDV	tRF-34-L85DMKYUYRLHIX	tRF-17-W18FLUF	tRF-46-OE0D58ZZJQJYSWRYVMD	tRF-17-W1X6L8L
tRF-16-K0SVRND	tRF-16-K53SW1B	tRF-36-P4R8YP9LON4VN1B	tRF-33-PY5P4PW3FJHPW	tRF-34-XRK4WO2F9IY9E2
	tRF-17-8SPOL52	tRF-35-EO7N987X1RJYSX	tRF-20-FP18LPMB	tRF-34-M2OSRNLNKSEKH9
	tRF-31-P4R8YP9LON4VD	tRF-19-URYRSIE2	tRF-39-YU4QUEVUUMHNU6E2	tRF-40-XENDBP1IUUK7VZ0R
	tRF-20-KS7SB1RH	tRF-21-UUK7VZ0RB	tRF-18-V29K9U0H	tRF-42-YQHQ9M739P8WQ0D52
	tRF-42-Y6IO43ZU2FYOB0152	tRF-29-34HWH3RXSIHM	tRF-36-VFYDNZDNB2Y0FDD	tRF-17-XU53F8L
	tRF-16-3VL8W1B	tRF-18-Z3M8ZL0D	tRF-21-UE3QHMY1B	tRF-23-5BF900BYDO
	tRF-16-3KXR3RB	tRF-21-MI7O3B1N0	tRF-28-2YU04DYJIOD3	tRF-26-MJ8F81KLB5D
	tRF-16-K8QJP1B	tRF-28-R81XDDZZ4YV	tRF-31-QKF1R3WE8RO80	tRF-27-945NB2NZW7L
	tRF-43-7O409Z7KZOSRI8WPD2	tRF-24-V6Z3M8ZL2J	tRF-26-OB1690PQR3E	tRF-50-JONY8B7OIQ1IBM4LV7U6
	tRF-37-QKF1R3WE8RO86J2	tRF-26-8DYDZDL9X1B	tRF-22-5OJIH6Z33	tRF-20-YV45EHBK
	tRF-42-YDU37Y93Y086Z34J2	tRF-20-J0DPZOIP	tRF-16-3QHMY1B	tRF-42-96DNBNHK4B8XVQN52
	tRF-17-KSP1852	tRF-19-I8W47WFD	tRF-33-V29K9UV36562DE	tRF-30-623K7SIR3DR2
	tRF-26-63U0EZY9X1B	tRF-16-8Z1MQ8E	tRF-26-U3XOUZDZN1B	tRF-26-IQ5O8LZ521B
	tRF-17-WJ9X0UF	tRF-16-3VWIQ1B	tRF-30-I363U0EZY9X1	tRF-20-K87SERM4
	tRF-22-Z3M8ZLSSP	tRF-30-J0DPZOIPD7VI	tRF-21-OB9ZFH69B	tRF-22-KS7SB1RHL
	tRF-35-L85DMKYUYRLHR0	tRF-28-K8HJ83ML5F02	tRF-39-PSQP4PW3FJIKE727	tRF-41-XENDBP1IUUK7VZ0RB
	tRF-17-RPM830K	tRF-45-NY8B7OIQ1IBM4LV7U6	tRF-19-I7JP8XI2	tRF-27-727OFIZ9WUJ
	tRF-36-QR535Z8LZBZFD90	tRF-16-489B3RB	tRF-32-EH623K7SIR3D4	tRF-17-8S68L52
	tRF-20-598LS7F6	tRF-36-JVK697BOJ8N981B	tRF-21-LN4Q1RV1B	tRF-41-8HM2OSRNLNKSEK51B
	tRF-35-INVDRI2Q2RJO01	tRF-25-3K7SIR3DR2	tRF-48-EBXNWD8NBNIED6VZ0OD2	tRF-17-W6VM4J2
	tRF-48-UIZMB3UEVPQOH7HLWXD2	tRF-16-8SPOL5D	tRF-39-XU53F85SI1LQ3RE2	tRF-21-2Q2RJO01B
	tRF-38-31QJ3KYUYRR6RBD2	tRF-49-I3Z9HMI8W47W1R7HFEE2	tRF-31-ZWRNU3KQ9MV1B	tRF-20-WVZ5EFOK
	tRF-20-K87SIRM1	tRF-32-7EMQ18Y3E7QNN	tRF-23-F9LKXNYQDE	tRF-16-WJ9X0UB
	tRF-34-9ZFH690M0RHBFV	tRF-38-YDU37Y93Y086Z3DO	tRF-21-WS7YRR500	tRF-17-WS3V2V4
	tRF-35-M2OSRNLNKSEK51	tRF-20-I8W47W1R	tRF-24-F9LKXNYQFP	tRF-22-WJ9X0UD3P
	tRF-27-EK45H5KQBFJ	tRF-22-ZFHIUNWV2	tRF-32-86V8WPMN1E8YN	tRF-35-EH623K7SIR3DR2
	tRF-32-L85DMKYUYRLH4	tRF-18-F9LKXN05	tRF-16-Q01LQKB	tRF-18-SRI8WPD2
	tRF-42-5ZLIJXDXXLHQIXKD2	tRF-32-7Y93Y086Z34J2	tRF-33-8NBNIED6VZ0OD2	tRF-37-LP21W3WB8US5652
	tRF-20-OL7XDXOM	tRF-16-3VLIE1B	tRF-21-YDZDL9X1B	tRF-25-HMI8W47W1R
	tRF-16-W1X6L80	tRF-23-8HYV45EH6	tRF-16-3KMB01B	tRF-30-JMRPFQ5DWXH9
	tRF-21-U0EZY9X1B	tRF-38-WD8NBNIED6VZ0OD2	tRF-25-R3VJ4ZZ526	tRF-32-JXDXXLHQIXKD2
	tRF-37-F9LKXNYQIUIVBNK	tRF-41-I9O9IOIQ5O8LZ521B	tRF-25-2IUIX1Q7O6	tRF-16-362VO00
	tRF-22-7PP9ZZ052	tRF-33-L85DMKYUYRLHDY	tRF-28-P4R8YP9LOND5	tRF-31-D890YBOZZ7NDD
	tRF-49-WXQZR44ER81XDDZZ4YE2	tRF-17-F9LKXNQ	tRF-22-8F81KLB52	tRF-40-2VR0PSR9593J2426
	tRF-30-897PVP941QKS	tRF-34-7N987X1RJYSXI2	tRF-22-UF04QZ452	tRF-27-HK4B8XVQN52
	tRF-16-8871K9D	tRF-24-5NB2NZW7HV	tRF-23-YUYRR6RBD2	tRF-17-KS01852
	tRF-25-1IBM4LV7U6	tRF-29-E7ZPVOH30W2R	tRF-21-987X1RJY0	tRF-43-Z6V6Z3M8ZLSSXUOLD2
	tRF-18-RPM830D4	tRF-32-PW5SVP9N15WVN	tRF-30-KQSX0DIJZ726	tRF-24-Q6J6K6UDE2
	tRF-23-QOH7HLWXD2	tRF-28-VPQOH7HLWXD2	tRF-29-987X1RJYSXI2	tRF-36-PW5SVP9N15WV7W0
	tRF-23-45H5KQBFD2	tRF-21-F9LKXNYQB	tRF-19-F9LKXNK2	tRF-30-VDRI2Q2RJO01
	tRF-36-EO7N987X1RJYSX0	tRF-32-KRIMUF04QZ452	tRF-24-7X1RJYSXI2	
	tRF-16-EO7N980	tRF-18-0RSSX7D2	tRF-18-86J8WPD4	hsa-miR-615-3p
	tRF-28-5BF900BY4D02	tRF-18-YSQSD2D2	tRF-25-J0DPZOIPD7	
	tRF-44-IEYU4QUEVUUMHNU6E2	tRF-19-K8YUBSI8	tRF-18-S7PVRSD2	

were in common between the MeT-5A untreated and exposed to 50 ug/ml FE fibers vs. JU77 untreated and exposed to 10 ug/ml FE fibers (Fig. 4D, Table 5). tRF-18-WKXU53DJ, tRF-48-9NZ6V6Z3M8ZLSSXUOLD2, tRF-19-OSM83OJX, tRF-22-7OFIZ9WUJ were the up-regulated tRNA-derived ncRNAs in common between the MeT-5A untreated and exposed to 50 ug/ml FE fibers vs. JU77 untreated and exposed to 50 ug/ml FE fibers (Fig. 4D, Table 5). If we compare MeT-5A exposed to 10 ug/ml FE fibers vs. MeT5A untreated and MeT-5A exposed to 50 ug/ml FE fibers vs. MeT5A untreated and JU77 exposed to 10 ug/ml FE fibers vs. JU77 untreated there was no compound in common (Fig. 4D, Table 5). tRF-28-727OFIZ9WUD2, tRF-18-51MLHVDB, tRF-19-86J8WP1Z, tRF-18-7X9PN5D5, tRF-23-1XDDZZ4YV, tRF-19-7X9PN5HJ, tRF-40-ZSE6YBY4HEJQY1B6, tRF-20-48Z8SSFK, tRF-31-897PVP941QKSD, tRF-18-J6K6UDV, and tRF-16-K0SVRND were the up-regulated tRNA-derived ncRNAs in common among MeT-5A exposed to 50 ug/ml FE fibers vs. MeT5A untreated and JU77 exposed to 10 ug/ml FE fibers vs. JU77 untreated and JU77 exposed to 50 ug/ml FE fibers vs. JU77 untreated (Fig. 4D, Table 5). Among all samples, no up-regulated compound there was in common (Fig. 4D, Table 5).

Surely, between miRNAs and tRNA-derived ncRNAs molecules, the latter were found to be the most deregulated among the samples compared.

The results of the differential expression analysis were reported in Sup. Table 1.

### Pathways analysis

Once we identified the differentially expressed miRNAs for each comparison, we tried to investigate the impact of their dysregulation in metabolic and signaling pathways by using MITHrIL^50^. MITHrIL fully exploits the topological information encoded by pathways when computing perturbation scores. Pathways are then modeled as complex graphs where each node is a biological element (protein-coding gene, miRNA, or metabolite), and each edge is an interaction between them^50^. Importantly, MITHrIL takes into account experimentally validated miRNA-mRNA interaction in order to predict their effects on biological pathways^50^.

The results demonstrated clear patterns of negative and positive perturbation scores involving 39 different pathways. Considering untreated JU77 vs. MeT-5A, 24 pathways showed a negative perturbation score while 15 pathways showed a positive perturbation score.

A similar trend was found in the case of the JU77 vs. MeT-5A exposed to 10 ug/ml FE fibers, indeed 23 pathways showed a negative perturbation score while 16 pathways showed a positive perturbation score. In fact, the Jak-STAT signaling pathway, the toll-like receptor signaling pathway, and the thyroid hormone signaling pathway showed a different trend between the samples mentioned above. In particular, in samples not exposed and exposed to 10 ug/ml FE fibers, the Jak-STAT signaling pathway and the thyroid hormone signaling pathway showed a negative and positive perturbation score, respectively. Both of these pathways reverse their score again in the samples exposed to the FE fiber concentration equal to 50 ug/ml. On the contrary, the toll-like receptor signaling pathway showed a positive perturbation score in the samples not exposed and a negative perturbation score in the samples exposed to both FE fibers concentrations.

**Figure 5:**
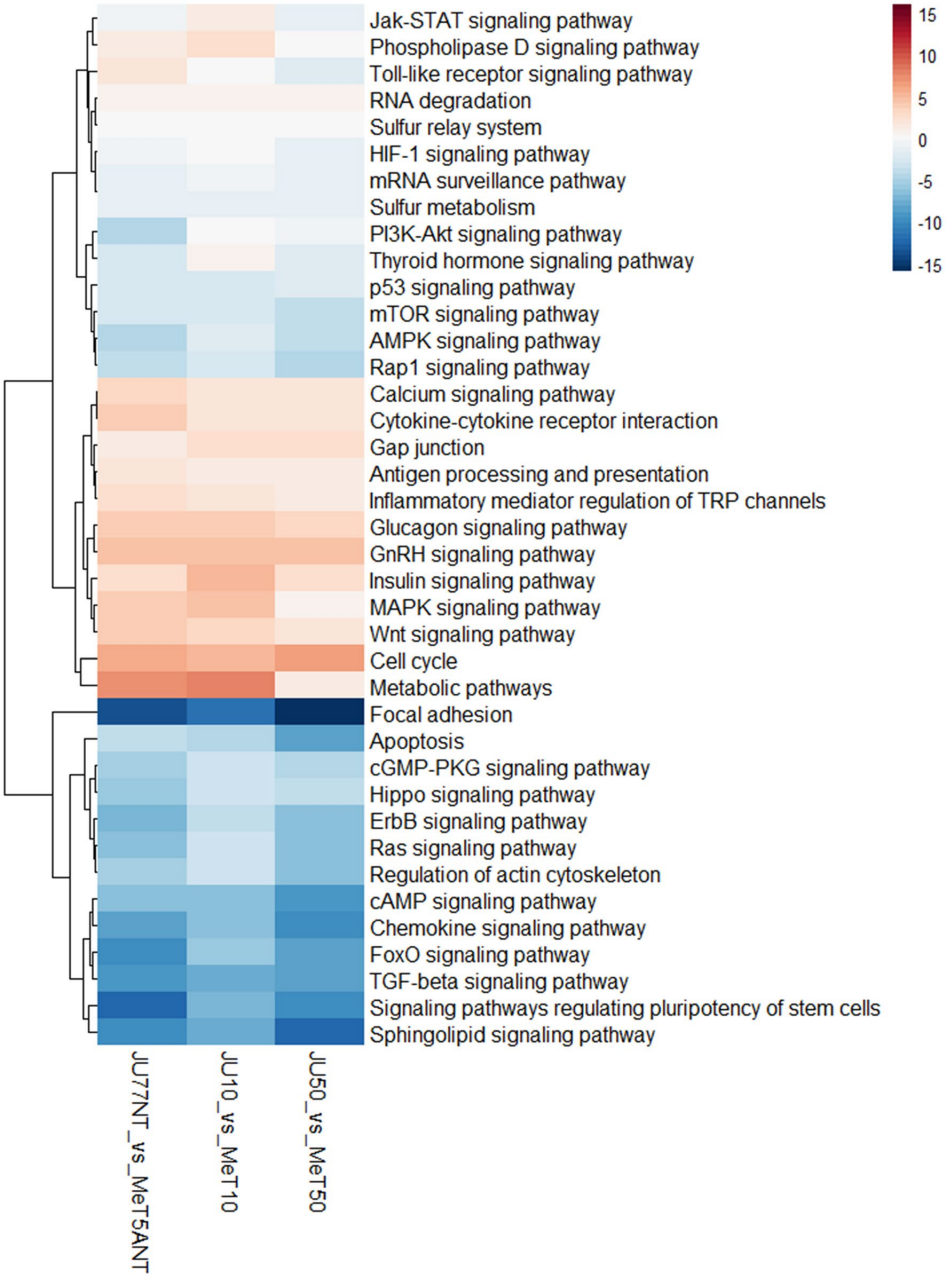
Heatmap showing the dysregulated pathways in JU77 (human malignant mesothelioma) vs. MeT-5A (human normal mesothelium) at the same concentration of FE fibers exposure by taking into account the differentially expressed miRNAs.

Considering the JU77 vs. MeT5A exposed to 50 ug/ml FE fibers, 26 pathways showed a negative perturbation score while 13 pathways showed a positive perturbation score. Comparing these results with the cells that did not undergo any exposure to FE fibers, the phospholipase D signaling pathway and the toll-like receptor signaling pathway showed a negative perturbation score after FE fibers exposure (Fig. 5).

**Figure 6:**
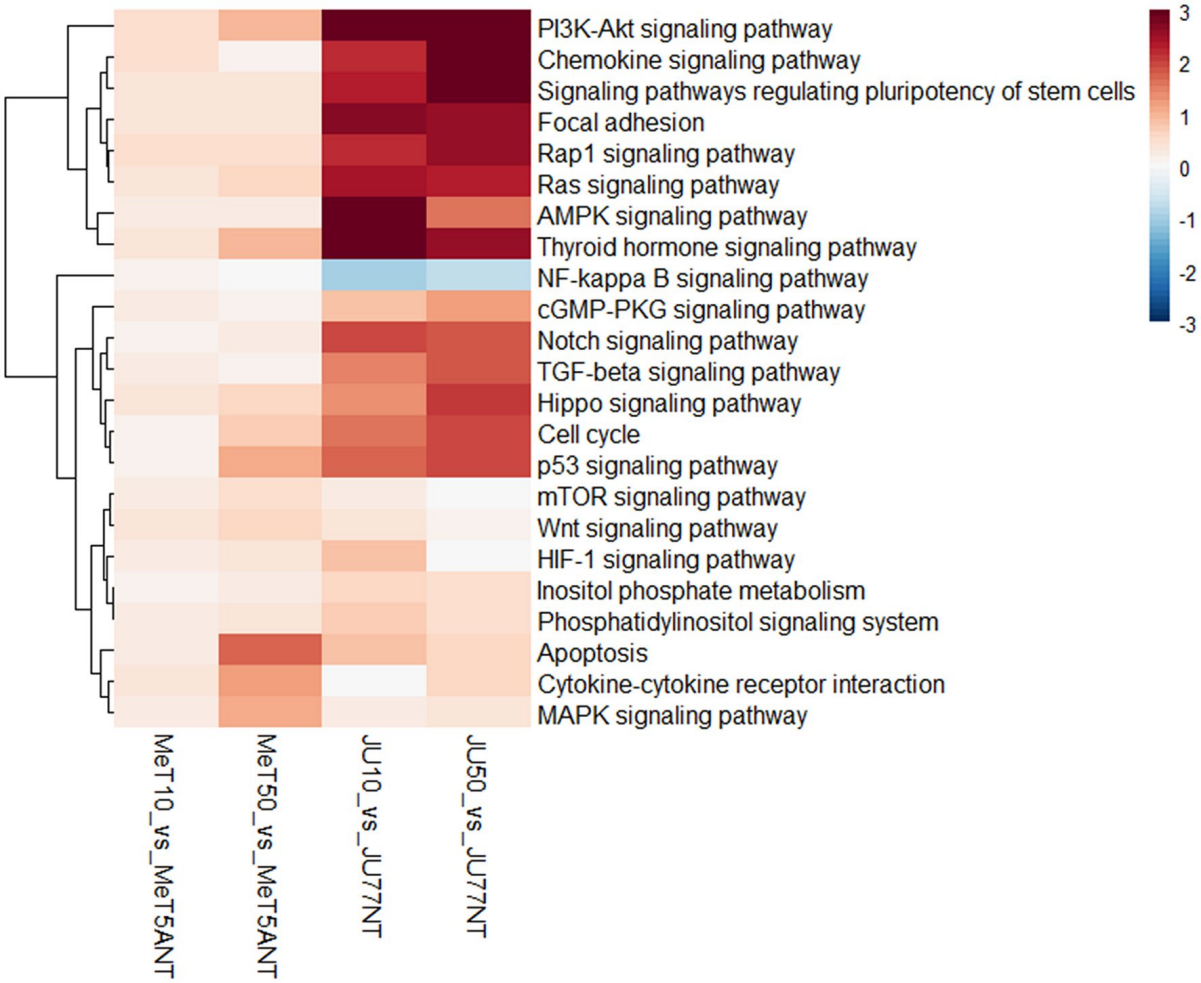
Heatmap showing the dysregulated pathways between untreated vs. FE fibers treated JU77 (human malignant mesothelioma) and MeT-5A (human normal mesothelium) by taking into account the differentially expressed miRNAs.

The pathway analysis performed between untreated vs. FE fibers treated JU77 and MeT-5A and the main impacted pathways showed clear patterns of positive correlations involving 23 different pathways. Only the NF-kappa B signaling pathway showed negative perturbation in the neoplastic samples (Fig. 6).

The results of the pathways analysis were reported in Sup. Table 2.

### Effects of fluoroedenite exposure in morphology and viability

**Figure 7:**
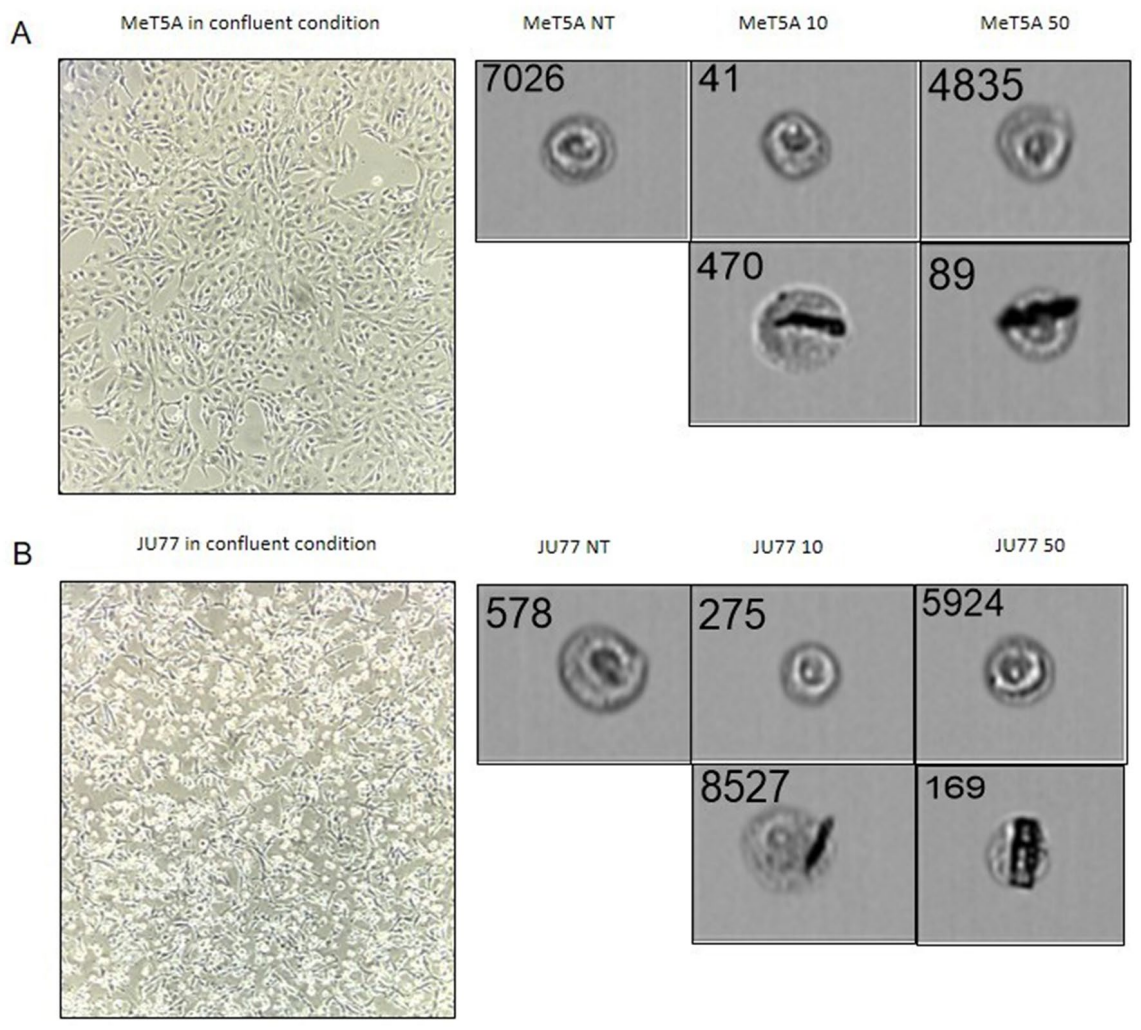
Morphology of cells in confluent condition immortalized through Nikon microscope Eclipse Ts2 (magnification 10x), and morphology of single cells immortalized through FlowSight Imaging Flow Cytometer after no exposure, exposure to 10 ug/ml FE fibers, and exposure to 50 ug/ml FE fibers (magnification 20x) for: A) MeT-5A (human normal mesothelium); B) JU77 (human malignant mesothelioma).

**Figure 8:**
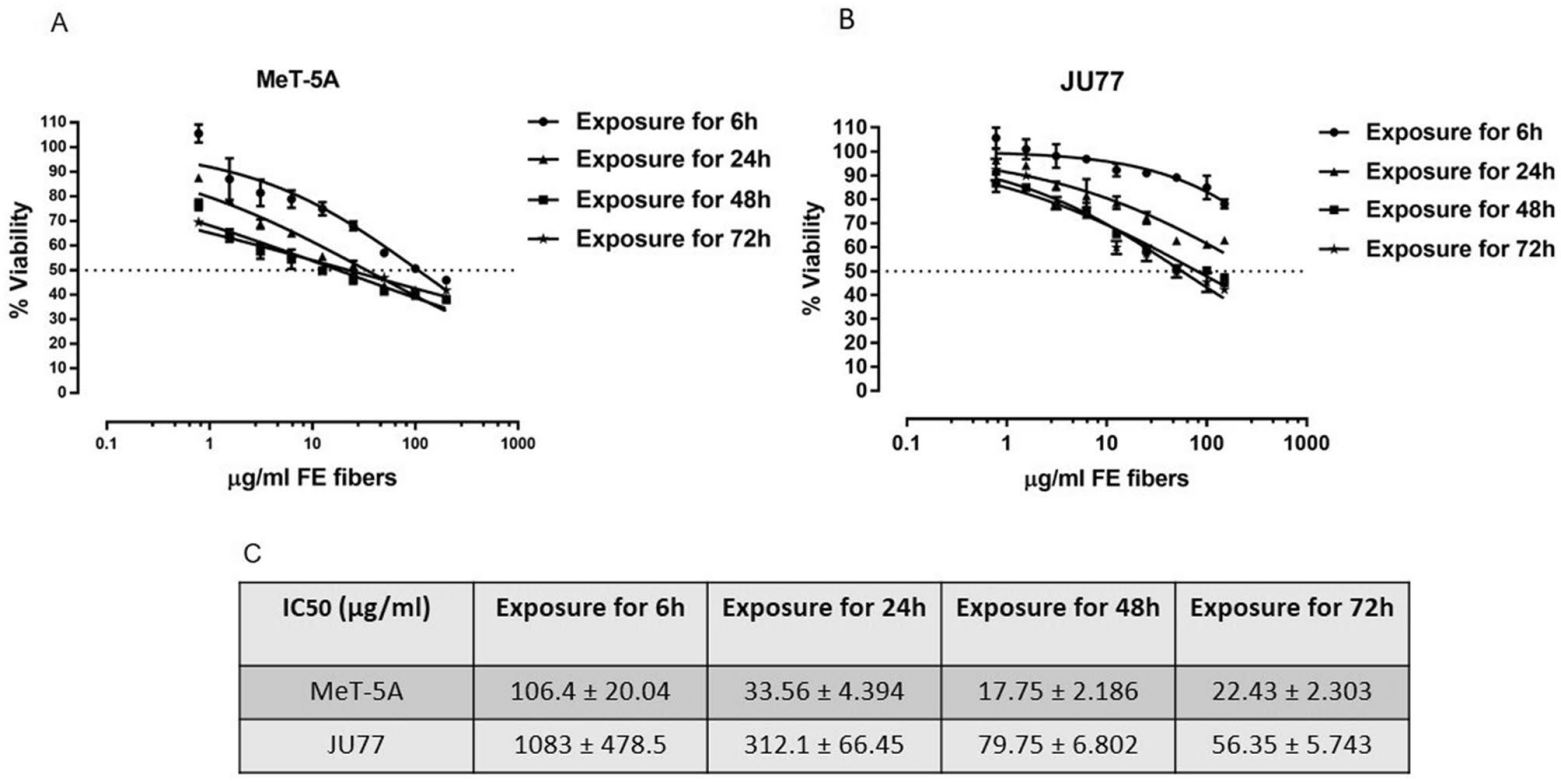
(**A**) Dose–response curves of MeT-5A (human normal mesothelium) with FE fibers from 200 to 0.78 μg/ml until 72 h of treatments; (**B**) Dose–response curves of JU77 (human malignant mesothelioma) with FE fibers from 200 to 0.78 μg/ml from 6 to 72 h of treatments; (**C**) IC50 values (μg/ml) for MeT-5A and JU77 cell lines.

MeT-5A are epithelial-like cells needle shaped that upon confluence develop a flattened shape. JU77 are epithelial-like cells spindle shaped with few vacuoles. Upon confluent condition assumed the epithelioid “cobblestone-like mat”. Both cell lines do not show evident morphological differences after exposure for 48 h to the different concentrations of FE fibers (10 and 50 µg/ml), with the exception of cells that come into direct contact with the fibers. In fact, these cells incorporate the fibers inside them, as shown in Fig. 7. Therefore, with the exception of the presence of fibers, neoplastic cells are not distinguished from healthy cells by cellular morphology. In accordance with Panzetta et al.^51^ morphology alone is not sufficient to discriminate malignant cells from benign cells. In addition, the in vitro viability of MeT-5A and JU77 from 6 to 72 h exposure to FE has been evaluated to examine the effects of FE fibers upon mesothelium and MM. The results showed that MeT-5A cells were more sensitive to FE fibers compared to JU77 cells, as shown in Fig. 8.

### Mesothelioma knowledge-network inference

**Figure 9:**
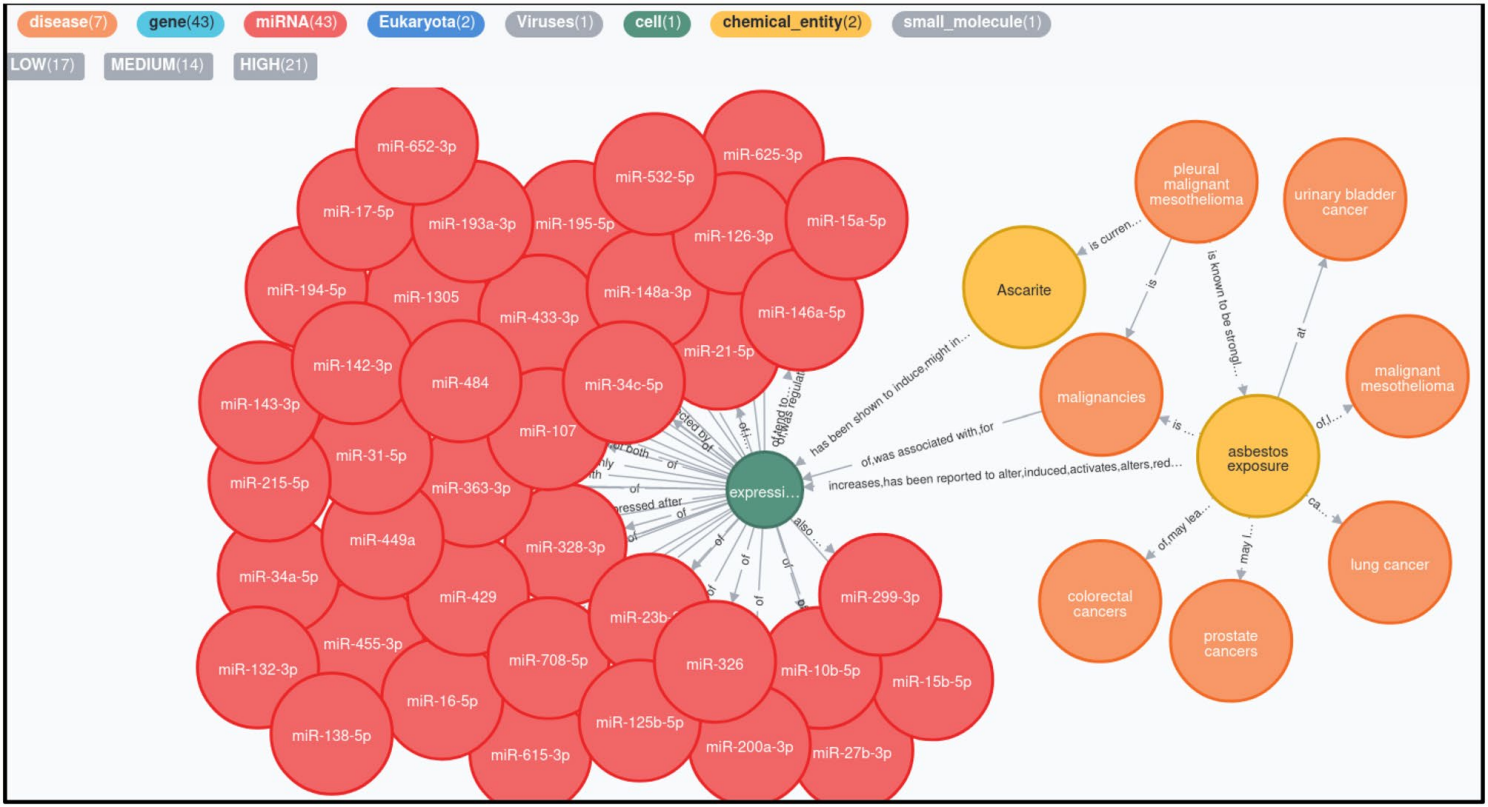
Mesothelioma-miRNAs network showing the relationships among pleural malignant mesothelioma disease and miRNAs derived from the PubMed Central full texts by using NetME tool. Such a network has been queried and displayed through Neo4j graph database and user interface.

NetME^47^ has been used to recover known interactions among malignant mesothelioma, asbestos, miRNAs, and other biological elements. For this purpose, we selected (through the NCBI APIs) the whole set of PubMed Central (PMC)^46^ documents related to the term “mesothelioma” in at least one of the following document sections: title, abstract, full text, references. The downloaded papers consist of 17.762 documents. Then, NetME generated the mesothelioma network as follows: (i) First, NetME converted the full text of the input documents into a list of nodes (biological entities) through literature databases and ontologies (Drug-Bank^52^, DisGenNET^53^, Obofoundry^54^, PubChem^55^); (ii) Next, an NLP model, based on Python SpaCy^56^ and NLTK^57^ libraries, has been executed to infer relations among nodes belonging to the same sentence (Si) or adjacent ones (S_i_, S_i+1_) of the same document. Such relationships represent disease-disease interactions, disease-gene interactions, gene regulations, molecular functions, etc. The network is composed of 2,468,187 nodes and 8,508,601 edges, and it is released in csv format to be loaded in Neo4j^48^, cytoscape^49^, or other graph visualization systems. The mesothelioma network was reported in Sup. csv files 1 and 2. Table 2 shows the most relevant sentences from 38 papers analyzed by NetME to infer the knowledge network. From these documents, it is clear how several miRNAs were involved in apoptosis, necrosis, and cell growth inhibition. Among these, the main miRNAs studied are miR-302b, miR-192, and miR-193a. The miR-193b is involved in the autophagy process while miR-1, miR-34a, miR-215, miR-16, miR-320a, and miR-21 seem to participate as oncogenes or oncosuppressors in malignant mesothelioma. To date, in literature, there are no papers that have analyzed tRNA-derived ncRNAs in malignant mesothelioma both induced and not induced by FE fibers. Data collected by NetME showed several research papers that demonstrated the correlation between asbestos exposure and different cancers. In fact, breathing asbestos fibers causes not only malignant mesothelioma, but also may lead to laryngeal cancer, prostate cancer, colorectal cancer, lung cancer, and ovarian cancer. Malignant mesothelioma and lung cancer have also been associated with exposure to carbon nanotubes. In Fig. 9, is shown the mesothelioma-miRNAs interactions network achieved by running the following cypher query against the whole mesothelioma network (stored in neo4j db): MATCH (n1:disease {name:”pleural malignant mesothelioma”})-[:*1.0.3]- > (n2:miRNA). Where, n1 (disease) and n2 (miRNAs) are the source and destination nodes, respectively. While, the term [:*1.0.3] selects all the paths (collections of nodes and edges into the network) from n1 to n2 having a minimum length of 1 and a maximum of 3. For example: path: (asbestos exposure)-[increases]- > (expression)-[of]- > (miR-107).

Each path has been inferred through the NetME syntactic analysis performed over the mesothelioma-based documents collected in PubMed Central.

## Discussion

Small RNA-Seq transcriptome profiling of healthy mesothelium and MPM in vitro has been evaluated to highlight the deregulated miRNAs and tRNA-derived ncRNAs and the different pathways involved in an aggressive cancer such as MPM.

The results showed that MeT-5A cells were more sensitive to FE fibers compared to JU77 cells and that morphology alone is not sufficient to discriminate malignant cells from benign cells. Certainly, a big difference was found in the number of deregulated miRNAs and tRNA-derived ncRNAs between tumor and non-tumor samples both exposed and not exposed to FE fibers. The common population of differentially expressed miRNAs and tRNA-derived ncRNAs between the two cell lines increased with the exposure to FE fibers. Though, the effect of exposure to FE fibers is more evident in the expression of miRNA and tRNA-derived ncRNAs in tumor samples than in non-tumor samples. Within the same cell line, the effect of exposure to FE fibers caused a dose-dependent increase in differentially expressed miRNAs and tRNA-derived ncRNAs. While the increase was noticeable in MeT-5A cells, in JU77 cells this was less pronounced. In the latter, in fact, the differentially expressed miRNAs and tRNA-derived ncRNAs were already many even in the neoplastic cells that had not undergone treatment with the cancer-causing fibers.

Several molecules aroused interest in their behavior in the various samples analyzed. Among them is a list of down-regulated and up-regulated miRNAs and tRNA-derived ncRNAs in common both between the two cell lines, and the different conditions of exposure with the FE fibers. While tRNA-derived ncRNAs have never been explored in the MPM, miRNAs have already been studied in this context. Several miRNAs are considered potential tumor suppressors in MPM. Expression of miR-15/16 family was consistently down-regulated both in MPM specimens and in cell lines and had tumor suppressor function in MPM^58^, in accordance with our results. miR-193a-3p is also considered a potential tumor suppressor in MPM^59^, indeed these miRNAs belonging to the group of mesomiRs (MM-associated miRNAs)^60,61^ could be considered a novel therapeutic approach for MPM.

In the present paper, we analyzed several pathways that are involved in the pathogenesis of malignant mesothelioma. It is very interesting to point out the strong perturbation scores involving the above-mentioned pathways in MPM vs*.* healthy mesothelial cells. Among these, there was the involvement of pathways that have important functions in inflammatory processes and in angiogenesis. Pathways that showed a different trend in healthy vs tumor samples at different FE fiber exposures were Jak-STAT, phospholipase D, toll like receptors, and thyroid hormone signaling pathways. Jak-STAT signaling pathway is involved in processes such as cell death, and carcinogenesis^62^. Phospholipase D activity is significantly increased in cancer tissues and cells, indicating that it plays a critical role in signal transduction, cell proliferation, and anti-apoptotic processes. In addition, phospholipase D is a downstream transcriptional target of proteins that contribute to inflammation and carcinogenesis such as Sp1, NF-kappa B, TCF4, ATF-2, NFATc2, and EWS-Fli^63^. Instead, toll like receptors have roles and functions in anti-cancer immunity and in tumor rejection^64^. Thyroid hormones not only regulate the physiological processes of normal cells but also stimulate cancer cell proliferation via dysregulation of molecular and signaling pathways^65^. Inflammation plays a central role since mesothelioma is a multicentric neoplasm, which originates from inflammatory foci. Inflammation has been correlated with cancer, enhancing the development of malignancies^66^. In particular, the chemokine and TGF-beta signaling pathways lead to acute and chronic inflammation, the latter resulting in several fiber-associated pulmonary and pleural diseases^14,67^. The inflammasome is responsible for the activation of inflammatory processes via multiple mechanisms^67^ that induce a process of cell death called pyroptosis, characterized by both apoptosis and necrosis. Cell death mechanisms and release of chemokines and cytokines may help cancer regress, and resist toxicity by fibers and cell growth^68^, in inflammasome-dependent and -independent pathways^69^. Indeed, apoptosis is a mechanism for removing cells with irreparable damage induced by FE fibers, causing genetic changes that predispose cells to a neoplastic transformation^70^. Specific signaling pathways such as sphingolipid, FoxO, and Hippo pathways are involved in many important signal transduction processes such as cell proliferation, and apoptosis^71–73^. Dysregulation of the Hippo signaling pathway is highly conserved by phosphorylating and inhibiting the transcription co-activators YAP and TAZ, key regulators of proliferation and apoptosis. On the contrary, dephosphorylated YAP/TAZ translocates into the nucleus and activates gene transcription through binding to TEAD family and other transcription factors. Such changes in gene expression promote cell proliferation and stem cell/progenitor cell self-renewal but inhibit apoptosis, thereby promoting tissue regeneration, and tumorigenesis^72^. An experimental model demonstrated the activation of YAP caused by ATG7 deletion^74^, which is an important transcription activator in malignant mesothelioma^75^. In our recent research, ATG7's high expression represents a promising prognostic tool for patients with MPM^76^, thus it would be interesting to explore if there is an inverse correlation between ATG7 and YAP in malignant mesothelioma. The leukocyte migration to the site of injury is led by chemokines. The first to be recruited to the site of injury are neutrophils, followed by monocytes, which differentiate into macrophages. Macrophages, once activated, are the main source of growth factors and cytokines which influence the local microenvironment. Mast cells, such as histamine, cytokines, proteases, and lipid mediators also contribute to inflammatory mediators^77^. In previous studies^14,78–80^, it was demonstrated the involvement of cytokines IL-18, IL-1beta, and NF-kappa B in the inflammasome activation process, suggesting that these immune-modulators are involved in the pathogenic mechanisms triggered by asbestos fibers. Acute and chronic inflammations often generate common molecular mediators^70^. The higher inflammation, with greater angiogenesis, causes the fatal outcome of the neoplasm. The release of angiogenic cytokines, including TGF-beta and VEGF, occurs during the angiogenesis process in the malignant mesothelioma progression^81,82^. In particular, VEGF represents the principal angiogenic cytokine involved in this cancer^81,83^, modulating also the development of pleural effusion and ascites through an increase in vascular permeability^84^. VEGF activation plays an essential role in increasing the survival of normal cells exposed to carcinogenic agents. The FE fibers are able to induce functional modifications of parameters with crucial roles in cancer development and progression^9^. The synthesis of VEGF and beta-catenin, two critical steps of epithelial cell activation pathways, are affected by the FE fibers exposure shown by an abnormal cellular status with upregulated cell activities and a risk of neoplastic transformation^85^. Furthermore, the influence of the FE fibers on cell motility has been demonstrated through a dysregulated and altered distribution of actin network^85^. Focal adhesion, which forms mechanical links between cytoskeleton and extracellular matrix (ECM), and adherens junction, result clearly involved in malignant mesothelioma pathogenesis. About that, our recent study, on FE exposure in lung fibroblasts, suggested an ECM remodeling that can give rise to profibrotic cellular phenotypes and tumor microenvironment^86^.

Specific signaling pathways have been found to be involved in malignant mesothelioma. Among these, Ras and p53, the commonly mutated genes associated with cancer, are rarely targeted in malignant mesothelioma^87^. Ras has not been found to be mutated in mesothelioma cell lines^88,89^. However, several receptor tyrosine kinase pathways have been shown to be activated in mesothelioma including the epidermal growth factor receptor (EGFR), insulin-like growth factor receptor (IGFR), and c-Met^90–92^, all of which activate Ras signaling. According to these results, several studies have already suggested that the PI3K-Akt pathway is hyperactivated in mesothelioma cell lines^87,93^, resulting in the gain or loss of function of its downstream proteins, 4E-BP1 and pS6, both crucial to the regulation of protein synthesis^94^. But, the prognostic role of the PI3K pathway in MPM is not yet defined^95^.

From papers analyzed by NetME to infer the knowledge network, it is clear that many miRNAs have the ability to positively or negatively influence various pathways involved in apoptosis, necrosis, cell cycle, and cell growth inhibition in malignant mesothelioma, in accordance with our experimental results. Among these,… Furthermore, several pathways, emerging from the literature involved in asbestos exposure, are in common with our data obtained from the expression profiles of miRNAs of healthy mesothelium and MPM in vitro both exposed and not exposed to FE fibers. Among the signaling pathways emerging from our in vitro analyses, we found that some of them are in common with the results processed by NetME. These include e.g. the NF-kappa B, p53, …. already discussed above. Surely, the computational analysis performed to establish the functional role of miRNAs in MPM pathogenesis has shown that these miRNAs can target genes playing a potentially key role in inflammatory processes, angiogenesis, and tumor cell development. Furthermore, miRNAs reach their biological impact by targeting multiple genes with similar biological effects^96^ thus, finding potential biomarkers is a very difficult aim that provides a lot of research work. However, surprising results emerged from the expression analysis of tRNA-derived ncRNAs, which have not yet aroused interest in the literature regarding their use as new potential biomarkers in malignant mesothelioma.

After this first preliminary work, the analysis will be followed by the validation of the most significantly deregulated miRNAs by RT-qPCR in an independent sample set. Subsequently, this screening of microRNAs and tRNA-derived ncRNAs will allow us to validate the most interesting and promising results as potential biomarkers for MPM. Our goal is the validation of the most promising results in patients chronically exposed to FE using the liquid biopsy, to provide a minimally invasive screening method for the secondary prevention of MPM. Early detection of circulating tumor biomarkers represents one of the most promising strategies to enhance the survival of cancer patients by increasing treatment efficiency^61,97^. Besides this large amount of data, further studies will be designed for the selection of the most significant miRNAs and tRNA-derived ncRNAs to test and validate their diagnostic potential in high-risk individuals.

## Methods

### Cell cultures

Human normal mesothelium (MeT-5A cells) and human malignant mesothelioma (JU77 cells) were obtained from the American Type Culture Collection (ATCC; Manassas, VA, USA). Both cell lines have been cultured in Roswell Park Memorial Institute 1640 (RPMI 1640) medium supplemented with 10% fetal bovine serum, 1% L-glutamine (Lonza; Walkersville, MD, USA), 1% non-essential amino acids solution (Gibco by Thermo Fisher Waltham; Massachusetts, USA), 1% penicillin/streptomycin (Lonza; Walkersville, MD, USA). The culture conditions were 37 °C in a humidified atmosphere with 5% CO_2_. The MeT-5A and JU77 cell lines were split 1:3 and 1:6 respectively, twice a week.

Cells in confluent condition were separated from the culture flask (SPL Life Sciences; Korea) using 0.25% trypsin in 2.21 mM EDTA solution (Corning; Manassas, VA, USA) and counted using Bürker chamber by Trypan Blue Stain 0.4% (Gibco by Life Technologies; NY, USA)^10^. The cells have been used for the experiments between the III and IV passages.

### In vitro treatments for RNA-Seq transcriptome profiling

FE fibers were collected from the Biancavilla quarry (Sicily, Italy). These were sterilized under UV light for 10 min, suspended in RPMI 1640 medium, and sonicated through Omni-Ruptor 4000 Ultrasonic Homogenizer (OMNI INternational Inc.; Kennesaw, GA, USA) for 10 min^10^. The stock solution was then diluted appropriately to obtain the different concentrations for in vitro treatments.

MeT-5A and JU77 were plated onto 100 × 20 mm Petri Dishes (Eppendorf; Hamburg, Germany) at the density of 1 × 10^6^ cells and 8.5 × 10^5^ cells, respectively. After 24 h of incubation, the medium of both cell lines has been removed and replaced with FE fibers solutions to final concentrations of 50 and 10 ug/ml. MeT-5A and JU77 cells grown in normal medium were used as controls. After 48 h from FE fibers exposure, pellets have been collected in duplicate.

After elimination of the supernatant, cells were harvested on ice by scraping in cold Dulbecco’s Phosphate-Buffered Saline (DPBS) (Corning; Manassas, VA, USA). Cells are then centrifuged at 0.2 × g for 5 min at 4 °C and suspended in 1 ml cold DPBS. The cell solution was transferred to Eppendorf tubes. Cells were centrifuged at 0.8 × g for 5 min at 4 °C and supernatant has been removed^10^. The samples have been stored to—80 °C until RNA isolation.

### RNA isolation

Total RNA containing small non-coding RNA was extracted from the cell lines using miRNeasy Mini Kit (QIAGEN; Venlo, Netherlands) according to the manufacturer’s recommended protocols (miRNeasy Mini Handbook 11/2020). The RNA was directly quantified by the absorbance ratio at λ = 260/280 nm through NanoDrop (ND 1000) UV–Vis spectrophotometer^10,98^. All samples were diluted at the final concentration of 50 ng/µl for the subsequent analysis.

### Small RNA-Seq

QIAseq miRNA library kit (QIAGEN, Hilden, Germany) has been used for small RNA-Seq library preparation following the manufacturer’s instructions. RNA samples were quantified and quality tested by Agilent 2100 Bioanalyzer RNA assay (Agilent Technologies, Santa Clara, CA). Libraries were then checked with both Qubit 2.0 Fluorometer (Invitrogen, Carlsbad, CA) and Agilent Bioanalyzer DNA assay or Caliper (PerkinElmer, Waltham, MA). Finally, libraries were prepared for sequencing and sequenced on single-end 150 bp mode on NovaSeq 6000 (Illumina, San Diego, CA).

### Small RNA-Seq analysis

Low-quality reads and adapters were trimmed using Trim Galore, which is a wrapper of FASTQC^99^ and Cutadapt^100^. After that, for miRNA analysis, trimmed reads were aligned onto the reference human genome (HG38 version) using HISAT2^101^. The generated SAM files were first converted into BAM files using Samtools^102^, and second, mapped reads were quantified by featureCounts (parameters: -d 14 –primary) using the GTF annotation file retrieved from miRBase^103^. All the above-mentioned steps for the miRNA analysis were performed using RNAdetector^104^ software. Concerning the tRNA-derived ncRNA analysis, we first generated an indexed transcriptome using Bowtie 2^105^ containing tsRNA and 5’ leader pre-tRNA sequences retrieved from tRFexplorer^106^. After that, trimmed reads were mapped on the indexed transcriptome using Bowtie 2 in order to filter them from the reads that may map on tRFs. Filtered mapped reads in SAM format were then converted into BAM files by Samtools^102^, and tsRNAs and 5’ leader pre-tRNA sequences were quantified by using bedtools (multicov -q 30). Unfiltered reads in FASTQ format that did not map on tsRNA and 5’ leader pre-tRNA sequences were given as input to MINTmap^107^ for identifying and quantifying reads associated with tRFs. After that, the raw counts obtained from the miRNA and tRNA-derived ncRNAs analyses were all harmonized together in order to have a single raw count matrix that can be used for the differential expression analysis. The raw count values were then normalized to scale the raw library sizes in Trimmed Mean of M values (TMM) by using edgeR^108^ and all miRNAs and tRNA-derived ncRNAs whose geometric mean of TMM values across all samples were less than one were removed from the analysis because they were non-expressed or expressed at a very low level. Finally, the filtered count matrix was used for the differential expression analysis using LIMMA^109^. miRNA and tRNA-derived ncRNAs with a |Log2FC|> 0.58 and an adjusted *p*-value (Benjamini–Hochberg correction) < 0.05 were considered differentially expressed. Finally, the impact of differentially expressed miRNAs on biological pathways was evaluated by using MITHrIL^50^. In this case, we selected all miRNAs with significant adjusted p-values without a Log2FC cutoff in order to evaluate also the impact of slightly differentially expressed miRNAs on biological pathways.

### Determination of dose–response curves and IC50

For the determination of dose–response curves, MeT-5A were plated onto 96-well plates at the density of 6 × 10^3^ cells/50 μl while JU77 were plated at the density of 4 × 10^3^ cells/50 μl. After 24 h of incubation, 50 μl of FE fibers solutions were added to the cell cultures in amounts corresponding to final concentrations of 200, 100, 50, 25, 12.5, 6.25, 3.12, 1.56, 0.78 μg/ml. Both cell lines grown in FE-free medium were used as controls. At each time point (6, 24, 48, 72 h of FE exposure) in cell culture, 10% MTT in DPBS has been added to each well. After 4 h of incubation, the lysis solution has been added to each well. The optical density was measured with an absorbance microplate reader at λ = 620 nm. For each sample, 3 replicates were performed. Cell viability was calculated as the percentage of viable cells exposed to FE fibers vs.*.* control cells no exposed as follows:

Cell viability (%) = [OD (Treatment) − OD (Blank)]/[OD (Control) − OD (Blank)] × 100. IC50 values have been calculated through the following equation:

[Inhibitor] vs. normalized response–Variable slope.

Results have been analyzed using PRISM GraphPad v. 7.00 and data were represented as the mean ± SD. An unpaired Student's *t*-test was used to compare data between the two groups. A value of p < 0.05 was considered statistically significant.

## Supplementary Information

Supplementary Information 1.

Supplementary Information 2.

Supplementary Information 3.

## Data Availability

Raw sequencing data will be deposited on NCBI SRA while processed data will be deposited on GEO". Raw data have been uploaded on SRA, BioProject ID: PRJNA812748.
